# Disarming Fungal Pathogens: *Bacillus safensis* Inhibits Virulence Factor Production and Biofilm Formation by *Cryptococcus neoformans* and *Candida albicans*

**DOI:** 10.1128/mBio.01537-17

**Published:** 2017-10-03

**Authors:** François L. Mayer, James W. Kronstad

**Affiliations:** Department of Microbiology and Immunology, Michael Smith Laboratories, University of British Columbia, Vancouver, British Columbia, Canada; Yonsei University

**Keywords:** capsule formation, cell wall, chitin, chitinase, interspecies interaction, melanization

## Abstract

Bacteria interact with each other in nature and often compete for limited nutrient and space resources. However, it is largely unknown whether and how bacteria also interact with human fungal pathogens naturally found in the environment. Here, we identified a soil bacterium, *Bacillus safensis*, which potently blocked several key *Cryptococcus neoformans* virulence factors, including formation of the antioxidant pigment melanin and production of the antiphagocytic polysaccharide capsule. The bacterium also inhibited *de novo* cryptococcal biofilm formation but had only modest inhibitory effects on already formed biofilms or planktonic cell growth. The inhibition of fungal melanization was dependent on direct cell contact and live bacteria. *B. safensis* also had anti-virulence factor activity against another major human-associated fungal pathogen, *Candida albicans*. Specifically, dual-species interaction studies revealed that the bacterium strongly inhibited *C. albicans* filamentation and biofilm formation. In particular, *B. safensis* physically attached to and degraded candidal filaments. Through genetic and phenotypic analyses, we demonstrated that bacterial chitinase activity against fungal cell wall chitin is a factor contributing to the antipathogen effect of *B. safensis*.

## IMPORTANCE

Pathogenic fungi are estimated to contribute to as many human deaths as tuberculosis or malaria. Two of the most common fungal pathogens, *Cryptococcus neoformans* and *Candida albicans*, account for up to 1.4 million infections per year with very high mortality rates. Few antifungal drugs are available for treatment, and development of novel therapies is complicated by the need for pathogen-specific targets. Therefore, there is an urgent need to identify novel drug targets and new drugs. Pathogens use virulence factors during infection, and it has recently been proposed that targeting these factors instead of the pathogen itself may represent a new approach to develop antimicrobials. Here, we identified a soil bacterium that specifically blocked virulence factor production and biofilm formation by *C. neoformans* and *C. albicans*. We demonstrate that the bacterial antipathogen mechanism is based in part on targeting the fungal cell wall, a structure not found in human cells.

## INTRODUCTION

Human fungal pathogens are responsible for over two million deaths annually (1, 2). To establish infections, these fungi rely on a set of specific virulence factors such as, for example, the production of a protective polysaccharide capsule, pigment biosynthesis, secretion of enzymes, biofilm formation, and morphological plasticity (3–5). Due to their eukaryotic nature and relatively close physiological similarity to human cells, pathogenic fungi are notoriously difficult to target for clinical therapy. In recent years, a novel concept in antimicrobial development has emerged to develop therapies that exclusively target microbial virulence factor elaboration instead of eradication of the pathogen itself (6). This approach can be seen as disarming pathogenic microorganisms and hence rendering them harmless and/or more susceptible to containment by antibiotics or the human immune system.

Some pathogenic fungi inhabit the environment as their natural niche, and they may infect humans (for example, by inhalation) to cause disease. Here, we hypothesized that other microorganisms sharing environmental reservoirs, especially bacteria, may have evolved antagonistic mechanisms directed against disease-causing fungi. Specifically, we were interested in identifying microbial antipathogen activities targeting fungal virulence factor production.

Two closely related fungal pathogens of humans, *Cryptococcus neoformans* and *C. gattii*, naturally occur in environmental niches, such as soil, trees, plants, and bird excreta (7). Infections occurring through inhalation can result in lung colonization and meningoencephalitis in immunocompromised patients and are often life-threatening. It was recently estimated that 223,100 individuals suffer from cryptococcal meningitis annually, with a mortality rate of over 80% (8). Regions in sub-Saharan Africa where the number of persons suffering from AIDS/HIV is extremely high have especially frequent incidences of cryptococcosis. Indeed, cryptococcal infections are responsible for 15% of all AIDS-related deaths worldwide (8).

The pathogenic *Cryptococcus* species form a dark-brown/black, cell wall-associated pigment termed melanin upon host infection (9). Melanin is a major virulence factor in these pathogens and has antioxidant properties (10). Mutants with defects in melanin production usually display reduced virulence *in vivo* (11–13). In this study, we isolated 40 microorganisms from *Cryptococcus*-inhabited niches and investigated their potential to block or dampen fungal melanin production. Dual-species interaction studies revealed that several bacterial species of the genus *Bacillus* had antimelanization activity. One bacterium in particular, *Bacillus safensis*, strongly inhibited cryptococcal melanization and melanin shedding in a contact-dependent manner. Importantly, *B. safensis* did not significantly impact overall fungal growth but did block expression of other key cryptococcal virulence factors, including capsule production and biofilm formation. Antivirulence activity was not limited to basidiomycetes, as *B. safensis* also potently inhibited virulence factor elaboration by the ascomycete *Candida albicans*, another major human fungal pathogen that is obligately associated with mammals (14). Through genetic and phenotypic analyses, we demonstrate that chitinase activity is a factor contributing to the destablization of the fungal cell wall by *B. safensis*.

## RESULTS

### A screen of environmental microbes identified bacteria that inhibit *C. neoformans* melanin production.

Our first objective was to investigate whether some of the microbes found in environmental niches that are coinhabited by *Cryptococcus* species may have antifungal activities. *C. neoformans* and *C. gattii* are found in the environment in soil and in bird droppings and are associated with trees and plants (7). We therefore assembled soil samples from Vancouver Island (a kind gift of Karen Bartlett, University of British Columbia) and plant samples from Vancouver (see Fig. S1A and S1B and Table S1 in the supplemental material). The soil samples were previously shown to be positive for the presence of *C. gattii*, and we hypothesized that this would increase our chances of identifying microbes with anticryptococcal activity (15). We isolated microbes from 24 different soil samples, covering nine different locations on Vancouver Island, and from two different plant samples from Vancouver. Between 1 and ~100 colonies grew per plate after 48 h at 30°C, and we chose 1 to 4 colonies per plate for further investigation. Selection criteria were based on an effort to include a variety of sampling locations and differences in colony appearance and color. In total, 40 isolates of diverse sizes and with predominantly rod-shaped morphologies were assembled (Fig. S1C).

10.1128/mBio.01537-17.1 FIG S1  Isolation of environmental microbes from soil and plant samples from the Vancouver area. (A) Geographic map of sampling sites. Circle colors refer to soil samples (blue) or plant samples (red) collected at the indicated sites. (The map was modified from http://www.atlas.gc.ca and contains information licensed under the Open Government license—Canada.) (B) Representative pictures of soil and plant samples used for the isolation of microbes. The plant shown is *Aucuba japonica*. (C) DIC microscopy images of the 40 environmental microbes that were analyzed in this study. Scale bar, 10 µm. Download FIG S1, TIF file, 6.4 MB. Copyright © 2017 Mayer and Kronstad. 2017 Mayer and Kronstad This content is distributed under the terms of the Creative Commons Attribution 4.0 International license.

10.1128/mBio.01537-17.9 TABLE S1  Environmental microbes isolated in this study. Download TABLE S1, DOCX file, 0.1 MB. Copyright © 2017 Mayer and Kronstad. 2017 Mayer and Kronstad This content is distributed under the terms of the Creative Commons Attribution 4.0 International license.

**FIG 1:**
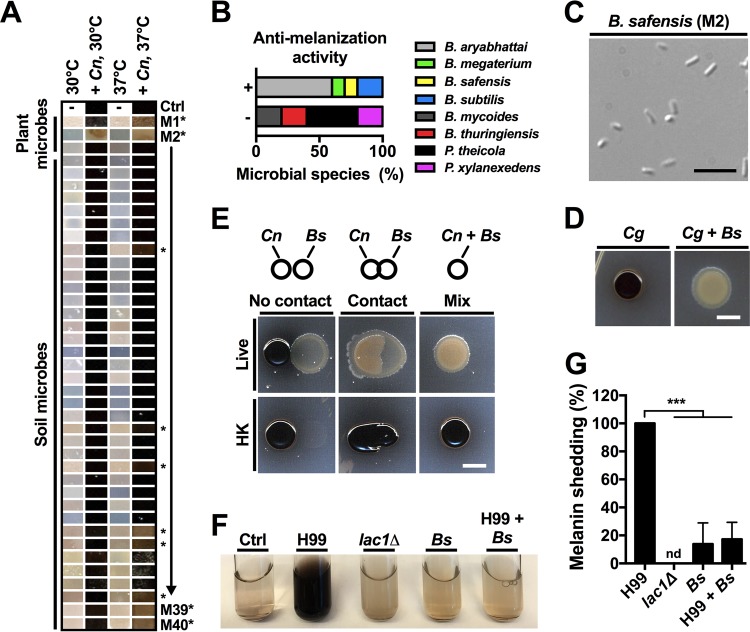
An environmental *Bacillus* strain inhibits cryptococcal melanization in a contact-dependent manner. (A) Colony color map (“melano-map”) of 40 environmental microbes, isolated from either soil or plants, following incubation on l-DOPA agar with or without *C. neoformans* wild-type strain H99. Fungal cells alone melanize and produce a black colony color. Cocultivation with some microbes results in impaired fungal melanization. Note that microbe M2 has strong antimelanization activity. *Cn*, *C. neoformans*; Ctrl, control; M1-M40, environmental microbe M1-M40. Stars indicate microbes with antimelanization activity. See Fig. S2A for additional information on assembly of the “melano-map.” The experiment was performed twice with similar results, and results from one experiment are shown. (B) Proportion of environmental microbes with activity against cryptococcal melanization. 16S rRNA sequencing revealed that all 10 microbes showing antimelanization activity belong to the *Bacillus* genus. For evaluation of microorganisms without antimelanization activity, five microbes were randomly selected and analyzed by 16S rRNA sequencing. *B. aryabhattai*, *Bacillus aryabhattai*; *B. megaterium*, *Bacillus megaterium*; *B. safensis*, *Bacillus safensis*; *B. subtilis*, *Bacillus subtilis*; *B. mycoides*, *Bacillus mycoides*; *B. thuringiensis*, *Bacillus thuringiensis*; *P. theicola*, *Pantoea theicola*; *P. xylanexedens*, *Paenibacillus xylanexedens*. (C) DIC microscopy image of the Gram-positive, rod-shaped bacterium *B. safensis*. Scale bar, 5 µm. (D) *B. safensis* (*Bs*) inhibits melanization of the *C. gattii* wild-type strain R265 on l-DOPA agar at 30°C. Scale bar, 5 mm. The experiment was performed twice, and representative images are shown. (E) Direct cell contact and live bacterial cells are required for the antimelanization activity. Strains were incubated on l-DOPA agar at 30°C for 48 h. HK, heat killed. Scale bar, 5 mm. The experiment was performed three times, and representative images are shown. (F) The antimelanization activity of *B. safensis* is recapitulated in liquid l-DOPA medium. Strains were incubated at 30°C and 180 rpm for 46 h. Ctrl, medium-only control. (G) Quantification of melanin shedding for cryptococcal cells grown in liquid l-DOPA medium alone or in the presence of *B. safensis* (see panel F). Quantification is based on OD_405_ measurements, and results are presented relative to the wild-type strain, H99, whose results have been set to 100%. nd, not detected. Results are the means + standard deviations (SD) of three independent experiments, each performed in triplicate. ***, *P* < 0.0001.

It has recently been proposed that targeting virulence factor production, rather than pathogen growth itself, may be a promising approach to prevent and treat disease while simultaneously reducing the likelihood of eliciting pathogen resistance (6). We therefore set out to determine whether any of the isolates had activity against cryptococcal production of the antioxidant pigment melanin. Melanin formation is a key virulence factor in cryptococci, and melanin-deficient mutants are usually strongly attenuated in virulence (11–13). We incubated each of the 40 isolates either individually or mixed with *C. neoformans* wild-type (wt) strain H99 on l-3,4-dihydroxyphenylalanine (l-DOPA) agar, a medium that induces strong melanin production by cryptococcal cells. We then manually selected a rectangular image of a region of the colony center (according to the scheme shown in Fig. S2A) and assembled each image fragment into a “melano-map” (Fig. 1A). None of the isolated microbes appeared to produce melanin under these conditions. Incubation with *C. neoformans* alone, and most of the fungus-microbe coincubations, resulted in black colonies, indicative of melanin production. Strikingly, some of the isolates appeared to inhibit fungal melanization, leading to production of colonies with a light brown to beige color. Isolate M2 in particular had a strong antagonistic effect on melanization, and the color of dual-species colonies was light beige (Fig. 1A and Fig. S2B). We next determined the identity of those isolates that demonstrated antimelanization activity by 16S rRNA sequencing. All of the isolates were identified as bacteria, and most of them belonged to the genus *Bacillus* (Fig. 1B). Indeed, all bacteria found to possess antimelanization activity were *Bacillus* spp., while five randomly selected isolates without activity included both *Bacillus* spp. and species from other genera (Fig. 1B). Isolate M2 was identified as *Bacillus safensis*, a Gram-positive, rod-shaped, motile bacterium found ubiquitously in soil (Fig. 1C).

10.1128/mBio.01537-17.2 FIG S2  Microbe M2 (*B. safensis*) has antimelanization activity against *C. neoformans*. (A) Scheme depicting the approach for assembly of the “melano-maps” shown in Fig. 1A and Fig. S4B. A *C. neoformans* colony is incubated on l-DOPA agar and turns black due to production of melanin. A rectangular region of the central part of the colony is manually selected, cut out, and used for assembly of the “melano-map.” (B) Microbe M2 inhibits cryptococcal melanization, while microbe M3 does not. The *C. neoformans lac1*Δ mutant lacking laccase was used for comparison. Strains were incubated on l-DOPA agar at 30°C for 48 h. Scale bar, 1 cm. The experiment was performed twice, and representative images are shown. (C) *C. neoformans* melanizes in *B. safensis*-spent l-DOPA medium. Cultures were grown at 30°C and 180 rpm for 48 h. Control, spent l-DOPA medium only. The experiment was performed twice, and a representative image is shown. (D) *B. safensis* blocks melanin shedding by *C. neoformans* on l-DOPA agar. The same images from Fig. 1E were photographed in front of a white background for better visualization of melanin shedding around the fungal colony. The arrow points to the zone of shed melanin. Scale bar, 5 mm. The experiment was performed three times, and representative images are shown. (E) *B. safensis* supernatant does not inhibit melanin production by *C. neoformans*. S1, S2, and S3 indicate concentrated supernatant from YPD-grown, PDB-grown, and YNB-LIM-grown cultures of *B. safensis*, respectively. Scale bar, 5 mm. Download FIG S2, TIF file, 4.2 MB. Copyright © 2017 Mayer and Kronstad. 2017 Mayer and Kronstad This content is distributed under the terms of the Creative Commons Attribution 4.0 International license.

### *B. safensis* blocks cryptococcal melanization and melanin shedding in a contact-dependent manner.

Due to the strong effect of *B. safensis* on fungal melanization, we decided to focus our further investigations on this bacterium. We first confirmed that *B. safensis* not only affected melanin formation by *C. neoformans* but also strongly inhibited melanization of *C. gattii* (Fig. 1D). We next verified that the observed antimelanization effect was not due to bacterial consumption of l-DOPA (which would potentially make less l-DOPA available for *C. neoformans* to synthesize melanin). To do so, we incubated *C. neoformans* in *B. safensis*-spent, filter-sterilized, l-DOPA medium. Fungal cells were still able to melanize, as indicated by a transition of the appearance of the medium from clear to black (Fig. S2C). These results suggested that the bacterial antimelanization activity was likely not simply due to competition for l-DOPA as a substrate but rather pointed to a specific antifungal mechanism.

To begin to elucidate how *B. safensis* impacted fungal melanin formation, we next investigated whether direct contact and bacterial viability were required for the observed melanin-inhibiting effect. We found that the inhibitory effect was observed only when the fungal and bacterial cells were spotted onto l-DOPA agar such that the cells were initially touching each other (Fig. 1E). Furthermore, heat-killed *B. safensis* did not exert antimelanization activity (Fig. 1E). We also noted that when the two microorganisms were spotted in contact with each other, bacteria appeared to swarm around *C. neoformans* colonies (Fig. 1E). These results indicated that both direct contact and live bacterial cells are required for the observed impact on melanin formation. Cryptococcal cells not only incorporate melanin pigment in their cell wall but also shed melanin into the cell periphery. This process contributes immune-modulatory activity during human infection. We noticed that fungal colonies coincubated with *B. safensis* on l-DOPA medium not only did not turn black but also appeared to shed much less melanin into the surrounding agar (Fig. S2D). We therefore next quantified a potential impact of the bacteria on the shedding of fungal melanin in liquid l-DOPA medium. Cultures incubated with *B. safensis* and *C. neoformans* did not turn black compared to the fungus-only control (Fig. 1F), and indeed, melanin shedding was strongly reduced upon coculture (Fig. 1G). Interestingly, we also detected shedding of melanin (or a melanin-like molecule) by *B. safensis* monocultures (Fig. 1G). *B. safensis* is known to possess laccases (the enzymes required for biochemical conversion of l-DOPA into pigment) (16); however, melanin shedding was relatively modest (Fig. 1G), did not translate into a change of color of the medium (Fig. 1F), and did not interfere with *C. neoformans* melanization (Fig. S2C).

*Bacillus* spp. are known to secrete over 160 small molecules (17). We therefore evaluated the possibility that *B. safensis* may secrete a small molecule (or a mixture of molecules) with antimelanization activity. To this end, we grew *B. safensis* in yeast extract-peptone-dextrose (YPD), potato dextrose broth (PDB; Difco), or yeast nitrogen base–low-iron medium (YNB-LIM) and used the concentrated supernatants to test for antimelanization activity. None of the three culture supernatants repressed melanin production (Fig. S2E). These results are consistent with a requirement for direct bacterial-fungal contact for suppression of *C. neoformans* melanization.

### Bacterial-fungal interactions impacted *C. neoformans* growth dynamics.

**FIG 2:**
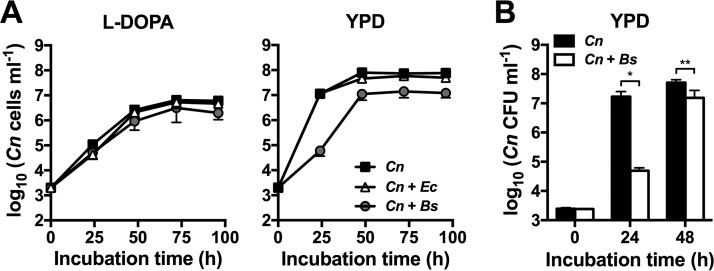
*B. safensis* does not affect the growth of *C. neoformans* in l-DOPA medium but delays fungal growth in complex YPD medium. (A) *C. neoformans* (*Cn*) was incubated in liquid l-DOPA or YPD alone or in the presence of either *E. coli* (*Ec*) or *B. safensis* (*Bs*). At the indicated time points, cell numbers were determined with a hemocytometer. Results are the means ± standard errors of the means (SEM) of at least two independent experiments. (B) Quantification of *C. neoformans* growth in YPD with or without *B. safensis*. Results are the means + SD of three independent experiments, each performed in duplicate. *, *P* < 0.01; **, *P* < 0.0001.

Having established that *B. safensis* has strong potency in inhibiting the production of a key cryptococcal virulence factor, we next determined whether the bacterium also affected fungal growth. Single-species incubations indicated that, as expected, *B. safensis* grew significantly faster in YPD than *C. neoformans* (Fig. S3). However, the two organisms had reached comparable cell densities after 72 h. Addition of the antibiotic gentamicin to YPD completely blocked bacterial growth without affecting fungal growth (Fig. S3). *B. safensis* grew even better in LB medium than in YPD; however, the LB medium did not sustain fungal growth (Fig. S3). Therefore, most of the fungus-bacterium coincubation studies described below were performed using YPD alone or YPD supplemented with gentamicin to inhibit *B. safensis* growth when necessary. Coincubation of *C. neoformans* with *B. safensis* had no effect on fungal growth dynamics in liquid l-DOPA medium compared to fungal monocultures (Fig. 2A). l-DOPA medium is nutrient limited, and we next assessed a possible impact of bacteria on fungal growth in complex YPD medium. Notably, the presence of *B. safensis* delayed fungal growth at early time points. At 24 h, fungal cell numbers in cocultures were ~180-fold reduced compared to fungal cultures incubated without bacteria. However, after 48 h, *C. neoformans* cell numbers in cocultures were only ~5-fold reduced compared to fungus-only control cultures (Fig. 2A). Coincubation with *Escherichia coli* had no effect on cryptococcal growth, pointing to differences in the interactions of both bacteria with this pathogen (Fig. 2A). To investigate the growth dynamics of *C. neoformans* upon interaction with *B. safensis* in YPD in more detail, we performed a similar bimicrobial incubation experiment and evaluated fungal growth using the enumeration of CFU. The results confirmed that fungal growth was significantly (~340-fold) reduced after 24 h of coincubation compared to fungus-only incubations. At 48 h, however, viable cell numbers had reached comparable values with a relatively modest, ~3-fold difference (Fig. 2B). In summary, *B. safensis* delays fungal proliferation at early time points but does not have a major impact on overall fungal growth after longer incubation times.

10.1128/mBio.01537-17.3 FIG S3  Gentamicin inhibits growth of *B. safensis*. Growth dynamics of *C. neoformans* and *B. safensis* in YPD and LB medium at 30°C and the block of bacterial growth by addition of gentamicin. Solid lines represent the means of results from two independent experiments, each performed in triplicate, and shaded areas represent the standard errors of the means. Download FIG S3, TIF file, 0.6 MB. Copyright © 2017 Mayer and Kronstad. 2017 Mayer and Kronstad This content is distributed under the terms of the Creative Commons Attribution 4.0 International license.

### *B. safensis* targets the *C. neoformans* cell surface to suppress melanin production.

**FIG 3:**
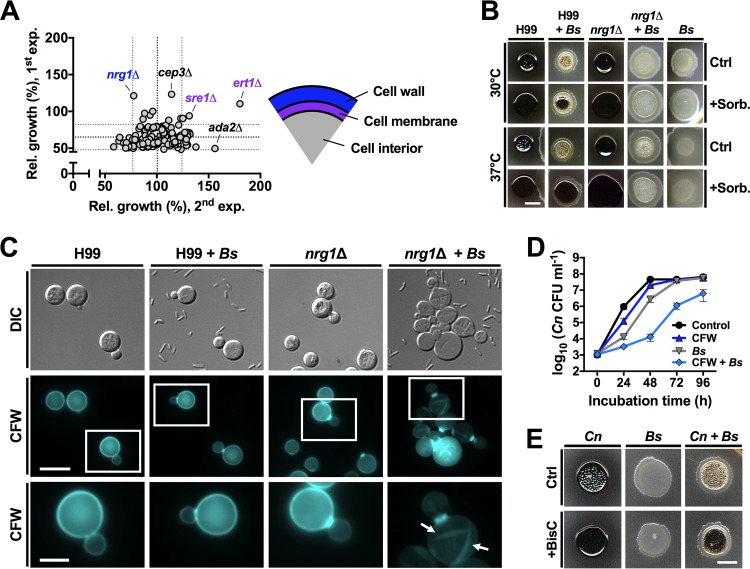
*B. safensis* targets the *C. neoformans* cell surface and bacterial chitinase activity contributes to the antimelanization effect during the dual-species interaction. (A) Individual coincubation of 154 different *C. neoformans* transcription factor (TF) mutants with *B. safensis* identified the fungal cell wall and cell membrane as possible targets. For each TF, two independent mutants were incubated in liquid l-DOPA medium with or without bacteria (308 TF strains in total) at 30°C for 5 days. Growth was determined via OD_405_ measurements. Results are plotted relative to the fungus-only control. Each circle represents the mean of the results for two independent mutants of the same TF. The black dotted lines and the gray dotted lines indicate the overall means of all values ± SD, respectively. The experiment (exp.) was performed twice, and TF mutant outliers (in at least one experiment) are indicated and color-coded according to previously published roles of these TFs in cell membrane and cell wall integrity (see scheme on the right). Rel. growth, relative growth of fungal cells in coculture versus monoculture, expressed in percentages. (B) Sorbitol bypassed *B. safensis*-mediated suppression of fungal melanization, and this effect was not observed in a *C. neoformans nrg1*Δ mutant. *C. neoformans* wild-type strain H99 or the *nrg1*Δ mutant was incubated alone or mixed with bacteria on l-DOPA agar with or without sorbitol for 48 h. Ctrl, control; Sorb, sorbitol. Scale bar, 5 mm. The experiment was performed twice, and representative images are shown. (C) Coincubation of *C. neoformans nrg1*Δ with *B. safensis* in YPD for 24 h resulted in fungal cell aggregation and abnormal cell wall chitin staining. White squares in the panels of the middle row indicate regions that are shown in a magnified view in the bottom row. Arrows point to abnormal, "scratch-like" CFW staining. DIC, differential interference contrast; CFW, calcofluor white. Scale bars indicate 5 µm (middle row) and 2 µm (bottom row). The experiment was performed twice, and representative images are shown. (D) *B. safensis* and CFW synergistically inhibit cryptococcal growth. CFU-based analysis of *C. neoformans* growth in YPD in presence of CFW or bacteria or a combination of the two was performed. Results are the means ± SD of two independent experiments, each performed in duplicate. (E) A chitinase inhibitor partially rescues *B. safensis*-mediated inhibition of fungal melanization on l-DOPA. Strains were incubated at 30°C for 48 h. Ctrl, control; BisC, bisdionine C. Scale bar, 5 mm. The experiment was performed three times, and representative images are shown.

Having demonstrated the capacity of *B. safensis* to inhibit *C. neoformans* melanization, we next sought to identify a potential mechanism. We reasoned that screening the recently established library of *C. neoformans* transcription factor (TF) mutants during coculture with *B. safensis* would provide clues to general fungal pathways or proteins potentially targeted by the bacterium (18). We performed the screen in liquid l-DOPA medium by culturing each of the 308 TF mutants (2 individual mutants per TF) alone or together with *B. safensis* for 5 days. Our original aim was to identify potential target candidates on the basis of evaluation of melanin production. However, the experiments were performed in 96-well plates and we obtained inconsistent results for melanin formation under those conditions. As an alternative and more general strategy, we examined the interactions using optical density (OD) measurements of fungal growth, and this approach provided a more robust evaluation of the response of *C. neoformans* to *B. safensis*. Specifically, when we compared final OD values at 405 nm (OD_405_) of dual-species cultures for each mutant with the OD_405_ values for fungal strains alone, we detected five mutants, namely, strains *nrg1*Δ, *cep3*Δ, *sre1*Δ, *ert1*Δ, and *ada2*Δ, with increased OD values during coculture compared to fungal monoculture in at least one of the two experiments performed (Fig. 3A). Subsequent testing of the five mutants revealed that none showed melanin defects on their own but that, similarly to the wt strain, melanization of each was inhibited by *B. safensis*.

We noticed that three of the five mutants (strains *nrg1*Δ, *sre1*Δ, and *ert1*Δ) had deletions in genes that have previously been shown to contribute to cell membrane and/or cell wall integrity (CWI) in *C. neoformans* (18, 19). We therefore hypothesized that *B. safensis* may target the fungal cell surface. To test this idea, we used l-DOPA agar supplemented with the cell membrane- and cell wall-stabilizing agent sorbitol and performed fungus-bacterium coincubation experiments. Strikingly, sorbitol rescued *B. safensis*-mediated inhibition of *C. neoformans* melanization, and this effect appeared to be temperature dependent, with more-pronounced melanization at 37°C (Fig. 3B). We verified that the sorbitol concentration used in these experiments did not negatively impact bacterial growth (Fig. S4A). We then investigated whether sorbitol may also bypass inhibition of fungal melanization mediated by the other bacterial species identified in our initial screen (Fig. 1A). With the exception of isolates M34 and M38 (both identified as *B. subtilis*; see Table S1), sorbitol bypassed inhibition of fungal melanization of all remaining bacteria (Fig. S4B). Next, we hypothesized that if *B. safensis* targets the fungal cell surface, melanization of the identified TF mutants (with defects in cell membrane/cell wall regulation) might not be rescued by sorbitol supplementation upon interaction with bacteria due to an additive negative effect on the cell surface. Indeed, sorbitol did not rescue melanin formation of the *nrg1*Δ and *sre1*Δ mutants under these conditions (Fig. 3B and Fig. S4C). The *ert1*Δ mutant, however, behaved like the wild type in this assay, and sorbitol did rescue melanin formation in the presence of *B. safensis*. These results indicated that the cell wall integrity (CWI) pathway may be involved in resistance to *B. safensis*-mediated fungal targeting. Consistently, sorbitol did not rescue melanization of specific CWI pathway mutants of *C. neoformans*, i.e., strains *hog1*Δ (20) and *mpk1*Δ (21), upon exposure to bacteria (Fig. S4C).

10.1128/mBio.01537-17.4 FIG S4  *B. safensis* targets the fungal cell surface. (A) The sorbitol concentration (1.5 M) that bypassed *B. safensis*-mediated inhibition of cryptococcal melanization does not affect bacterial growth. Bacteria were grown at 30°C and 180 rpm for 24 h, and the OD_600_ was determined. Ctrl, medium-only control; ns, not significant. Results are the means + SD of three independent experiments. (B) With exception of isolates M34 and M38, sorbitol bypasses the antimelanization activity of all microbes shown to inhibit melanin formation by *C. neoformans* in this study. Strains were incubated on l-DOPA agar, and a “melano-map” was assembled according to Fig. S2A. The experiment was performed twice with similar results, and results from one experiment are shown. (C) Sorbitol does not rescue melanization by *C. neoformans* mutants defective in the response to cell membrane and cell wall-directed stresses. Scale bar, 5 mm. The experiment was performed twice, and representative images are shown. (D) A combination of *B. safensis* and Congo red (CR) does not result in synergistic inhibition of *C. neoformans*. CFU-based analysis of *C. neoformans* growth in YPD in the presence of CR or bacteria or a combination of the two was performed. Note that the data for the *C. neoformans* control and for *B. safensis* are the same as those described for Fig. 3D and have been included for comparison. Results are the means ± SEM of two independent experiments, each performed in duplicate. (E) Quantification of CFW staining for *C. neoformans* cells incubated with or without *B. safensis* in YPD at 30°C. MFI, mean fluorescence intensity. Results are the means + SD of 20 cells analyzed. ***, *P* < 0.0001. (F) DIC and fluorescence microscopy images of *C. neoformans* cells incubated with or without *B. safensis* and stained with CFW. The arrow points to a fungal cell with strong CFW staining. Scale bar, 2 µm. (G) The *C. neoformans nrg1*Δ mutant is more susceptible to *B. safensis* than the wild-type strain. CFU-based analysis of the indicated fungal strains grown with or without bacteria in YPD was performed. Results are the means + SD of three independent experiments, each performed in duplicate. **, *P* < 0.001. (H) Coincubation of *C. neoformans* with *B. safensis* results in altered FM4-64 membrane-staining dynamics. In fungal cells incubated alone, FM4-64 is internalized and stains the vacuolar membrane, while the presence of bacteria leads to a diffuse, punctate staining pattern. Note that FM4-64 also stains punctate structures within the bacterial cells. Scale bar, 5 µm. (I) Chitinase does not inhibit *C. neoformans* or *C. gattii* melanization. Ctrl, control; Chit, chitinase; *Bs*, *B. safensis*. Scale bar, 5 mm. Download FIG S4, TIF file, 7.7 MB. Copyright © 2017 Mayer and Kronstad. 2017 Mayer and Kronstad This content is distributed under the terms of the Creative Commons Attribution 4.0 International license.

Due to the strong defect in melanization of the *nrg1*Δ mutant during interaction with *B. safensis*, we next investigated single-species and dual-species fungus-bacterium cultures of this particular mutant in more detail. Surprisingly, *nrg1*Δ cells exposed to bacteria for 24 h in YPD displayed aberrant cell morphologies, with cell surfaces that appeared to have been “scratched” (Fig. 3C). Staining with the chitin-binding dye calcofluor white (CFW) confirmed that *nrg1*Δ cells exposed to *B. safensis* had altered cell surface morphologies (Fig. 3C). The “scratch”-like phenotype is highly reminiscent of a phenotype previously observed for the *C. neoformans* chitin synthase mutants *chs3*Δ and *csr2*Δ (22, 23). These results therefore suggested that *B. safensis* targets the fungal cell wall at the level of chitin. To test this possibility, we next coexposed *C. neoformans* to bacteria and CFW. We reasoned that fungal growth would be synergistically inhibited if *B. safensis* indeed targets fungal chitin. Consistent with that assumption, a time course analysis of growth revealed that coincubation of fungal cells with either CFW or *B. safensis* modestly inhibited *C. neoformans* growth compared to the fungus-only control. Coapplication of bacteria and CFW, however, led to a synergistic inhibitory effect on fungal proliferation (Fig. 3D). These findings were specific for chitin because an analogous experiment using Congo red (a dye that binds cell wall β-1,3-glucan) did not demonstrate an impact on fungal growth (Fig. S4D). We also noticed that some wild-type *C. neoformans* cells in dual-species cultures with *B. safensis* appeared to stain slightly more strongly for cell wall chitin using CWF, and we confirmed this observation quantitatively (Fig. S4E and S4F). The strong phenotype of the *nrg1*Δ mutant upon coincubation with *B. safensis* prompted us to examine its growth in the presence of bacteria. Interestingly, an assay based on enumeration of CFU revealed that cells of the *nrg1*Δ mutant were significantly more susceptible to *B. safensis* after 48 h of coincubation than those of the wild-type strain (Fig. S4G). To test whether *B. safensis* may also impact the fungal cell membrane, as suggested by the identification of some of the TF mutants with defects in membrane function, we next used the membrane-staining dye FM4-64 to stain *C. neoformans* cells from single-species and dual-species cultures. While FM4-64 stained the vacuolar membrane of fungus-only cultures in a distinctive ringlike fashion, fungal cells from cocultures displayed a diffuse staining pattern across the entire cell (Fig. S4H). These results indicate that *B. safensis* may interfere with the architecture of both the cell wall and the cell membrane.

On the basis of the similar phenotypes of *B. safensis*-exposed *nrg1*Δ cells and chitin synthase mutants and of the synergistic effect of the chitin-binding compound CFW on the growth of *C. neoformans* in the presence of bacteria, we hypothesized that bacteria may target fungal cell wall chitin by chitinase activity. We therefore determined the effect of the chitinase inhibitor bisdionine C (BisC) on fungal melanization in dual-species cultures. Consistent with our hypothesis, BisC rescued *C. neoformans* melanization at least partially (Fig. 3E). Interestingly, commercially acquired chitinase did not prevent melanization of *C. neoformans* in these experiments (Fig. S4I). This result may have been due to the need for *B. safensis* to contact the fungal surface to impact chitin and also to the fact that the bacterium may produce more than one factor to exert an influence on melanization. Nonetheless, our results obtained with CFW and BisC suggest that bacterial chitinase activity is a factor contributing to the observed inhibition of cryptococcal melanin production.

We next reasoned that chitinase activity of *B. safensis* may release increased levels of chito-oligomers from fungal cells. Wheat germ agglutinin (WGA) specifically binds chito-oligomers (24), and we used it to stain *C. neoformans* cells following incubation with or without *B. safensis*. As predicted, we detected a modest but significant increase in WGA-staining intensity for fungal cells exposed to bacteria compared to controls (Fig. S5A and S5B). We also noted that the cells of *C. neoformans* showed a significant increase in size following dual-species interaction (Fig. S5C). Overall, these results contribute to the conclusion that *B. safensis* targets the fungal cell wall at least in part through chitinase activity, resulting in destabilization of cell wall architecture and changes in morphology and ultimately leading to improper virulence factor expression.

10.1128/mBio.01537-17.5 FIG S5  Coincubation with *B. safensis* induces the formation of larger *C. neoformans* cells and modestly enhances staining of fungal cell wall chitin and chito-oligomers. (A) Quantification of CFW and WGA staining of *C. neoformans* cells following growth in YPD with or without *B. safensis*. CFW, calcofluor white (stains chitin); WGA, wheat germ agglutinin (stains chito-oligomers). MFI, mean fluorescence intensity. Results are the means + SD of 20 cells analyzed per condition. *, *P* < 0.01; ***, *P* < 0.0001. (B) DIC and fluorescence microscopy images of *C. neoformans* cells grown with or without *B. safensis* and stained with CFW and WGA. Scale bar, 2 µm. (C) Quantification of *C. neoformans* cell size following growth in the absence or presence of *B. safensis*. Results are the means + SEM of 20 cells analyzed. **, *P* < 0.001. Download FIG S5, TIF file, 2.9 MB. Copyright © 2017 Mayer and Kronstad. 2017 Mayer and Kronstad This content is distributed under the terms of the Creative Commons Attribution 4.0 International license.

### *B. safensis* blocks *C. neoformans* virulence factor production.

**FIG 4:**
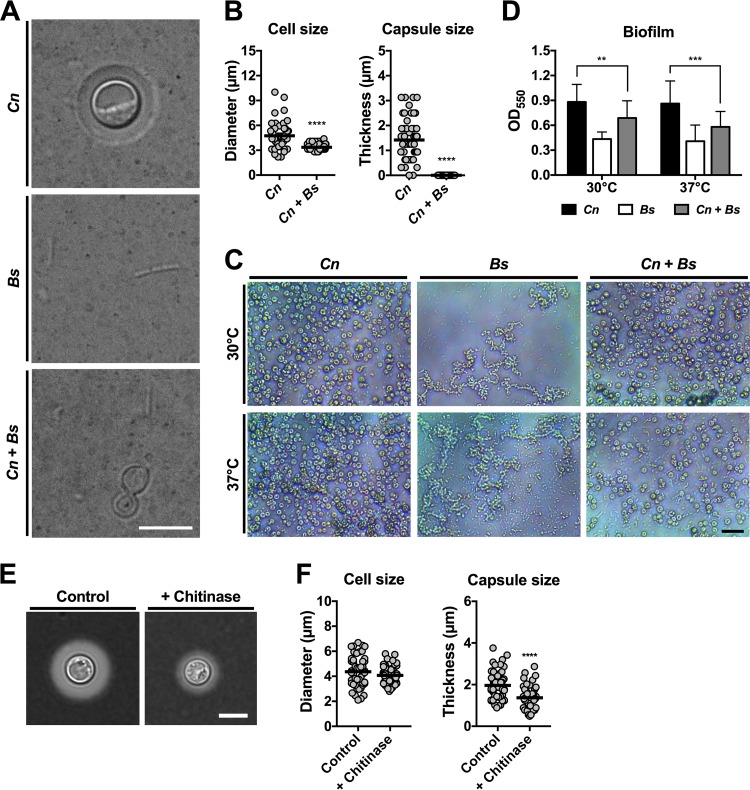
*B. safensis* inhibits *C. neoformans* capsule production and biofilm formation. (A) DIC microscopy images of India ink-stained fungal and bacterial monospecies or dual-species cultures grown under capsule-inducing conditions (10% FCS). Note that the fungal cells did not produce capsule in the presence of bacteria. Scale bar, 5 µm. (B) Quantification of *C. neoformans* cell and capsule size following incubation under capsule-inducing conditions with or without *B. safensis*. The experiment was performed twice, and at least 25 cells were analyzed per experiment and condition. Circles indicate individual data points from both experiments, and the black bar indicates the mean. ****, *P* < 0.0001. (C) Microscopy images of biofilms formed by *C. neoformans* or *B. safensis* or a coculture of the two. Note that cocultivation resulted in reduced fungal cell numbers compared to fungal monoculture, especially at 37°C. Scale bar, 20 µm. (D) Quantification of fungal and bacterial biofilm formation by crystal violet staining. Results are the means + SD of four independent experiments, each performed at least in triplicate. **, *P* < 0.01; ***, *P* < 0.001. (E) DIC microscopy images of India ink-stained *C. neoformans* cells grown under capsule-inducing conditions (CIM) with or without 100 µg ml^−1^ chitinase. Scale bar, 5 µm. (F) Quantification of *C. neoformans* cell and capsule size following incubation with or without chitinase. The experiment was performed twice, and results from one experiment are shown. A total of 50 cells were analyzed per condition. The black bar indicates the mean. ****, *P* < 0.0001. Scale bar, 5 µm.

Besides melanin synthesis, cryptococcal cells also produce other disease-relevant virulence factors. These factors include production of a protective and immune-modulatory polysaccharide capsule and biofilm formation (5). Given the strong bacterial impact on fungal melanization, we next investigated whether *B. safensis* may also affect expression of these other fungal virulence factors. Strikingly, coincubation of *C. neoformans* with bacteria in 10% fetal calf serum (FCS; a capsule-inducing medium) resulted in a complete block in fungal capsule formation (Fig. 4A). Though the fungal cells were modestly reduced in overall cell size, no capsule could be detected in coincubations compared to fungal monocultures (Fig. 4B).

Because some of the polysaccharides present in the capsule are also part of the cryptococcal biofilm matrix (25), we hypothesized that *B. safensis* may also negatively impact fungal biofilm formation. Indeed, when we exposed *C. neoformans* to *B. safensis* under biofilm-inducing conditions at a ratio of 1:1, we detected a significant reduction in the overall level of biofilm formation of coincubations compared to incubations performed with a single fungal species (Fig. 4C and D). This effect was temperature dependent, as overall biofilm formation was more strongly reduced at 37°C than at 30°C. It should be noted that in this experiment the actual level of fungal biofilm formation was probably even lower than indicated, as biofilm formation was measured for the mixture of fungal and bacterial cells. On the basis of our previous findings of a possible role of *B. safensis* chitinase activity during interaction with *C. neoformans*, we next investigated whether addition of exogenous chitinase alone might impact capsule formation by *C. neoformans*. Consistent with previously published findings, chitinase significantly inhibited capsule formation (Fig. 4E and F) (24). Overall, these findings indicate that *B. safensis* has strong potency in preventing *C. neoformans* capsule formation and significantly reduces fungal biofilm production.

### Hypha formation of the human-associated pathogen *C. albicans* is inhibited during interaction with *B. safensis.*

Having demonstrated that *B. safensis* potently inhibits the elaboration of cryptococcal virulence factors without having a substantial antagonistic impact on growth, we next determined whether the bacterium exhibited anti-virulence factor activity during interactions with other fungal pathogens. Specifically, we performed bimicrobial interaction studies with another major human fungal pathogen, *C. albicans*. In contrast to *C. neoformans* and *C. gattii*, *C. albicans* is not found in the environment but is obligately associated with warm-blooded animals such as humans (14). One of the main *C. albicans* virulence factors is the capacity to transition between a round yeast form and an elongated hyphal form (the yeast-to-hypha transition) (26). We therefore focused on a potential effect of *B. safensis* on *C. albicans* hypha formation. Coincubation of bacterial and fungal cells under conditions that induced yeast growth (YPD, 30°C) did not affect *C. albicans* cell morphology or proliferation compared to fungus-only cultures (Fig. S6A). However, under hypha-inducing conditions (10% FCS, 37°C), *B. safensis* strongly inhibited *C. albicans* hypha formation compared to control cultures with the fungus alone (Fig. S6A). We estimated the levels of yeast and hypha production under these conditions by determining the wet weights of cell pellets following overnight sedimentation at 4°C and centrifugation. The wet weights of pellets of yeast-phase cells from monospecies and dual-species cultures were not significantly different. However, significantly more hyphal mass was formed by *C. albicans*-only cultures than by dual-species cultures, thus confirming our microscopic examination (Fig. S6B). It should be noted that the evaluation of *C. albicans* morphologies was semiquantitative and may have been affected by carryover effects of the presence of bacterial cells following sedimentation and centrifugation of the dual-species culture. Due to the relatively small size of *B. safensis* cells compared to *C. albicans* yeast and hyphal cells, however, these effects should have been negligible. To investigate whether *B. safensis* also affected *C. albicans* hypha formation on solid media, we incubated fungal cells alone or mixed with *B. safensis* or *E. coli* on water agar supplemented with 10% FCS. The fungal cells incubated alone showed strong filamentation following a 9-day incubation time, and the presence of *E. coli* did not affect this process (Fig. S6C). However, consistent with our previous observations, *B. safensis* potently inhibited hypha formation under these conditions (Fig. S6C).

10.1128/mBio.01537-17.6 FIG S6  *B. safensis* inhibits *C. albicans* hypha formation. (A) Coincubation of *C. albicans* with *B. safensis* results in strongly reduced fungal filamentation in 10% FCS. Note that the yeast phase of fungal growth (YPD) does not appear to be affected by the presence of bacteria. Scale bar, 10 µm. (B) Semiquantitative evaluation of the bacterial impact on *C. albicans* yeast and hypha formation. The cultures incubated as described for panel A were centrifuged, the supernatant was discarded, and the respective pellet wet weights were determined. ns, not significant. Results are the means + SD of three independent experiments, two performed in triplicate and one performed as a single analysis. *, *P* < 0.001. (C) *B. safensis*, but not *E. coli* (*Ec*), inhibits *C. albicans* hypha formation. The indicated strains or strain mixtures were incubated on solid water agar supplemented with 10% FCS at 30°C for 8 to 9 days. White squares in the upper row panels indicate regions that are shown in a magnified view in the bottom row. Scale bar, 1 cm. The experiment was performed three times, and representative images are shown. Download FIG S6, TIF file, 4.5 MB. Copyright © 2017 Mayer and Kronstad. 2017 Mayer and Kronstad This content is distributed under the terms of the Creative Commons Attribution 4.0 International license.

### *B. safensis* attaches to *C. albicans* hyphae and suppresses fungal adhesion capacity and *de novo* biofilm formation.

**FIG 5:**
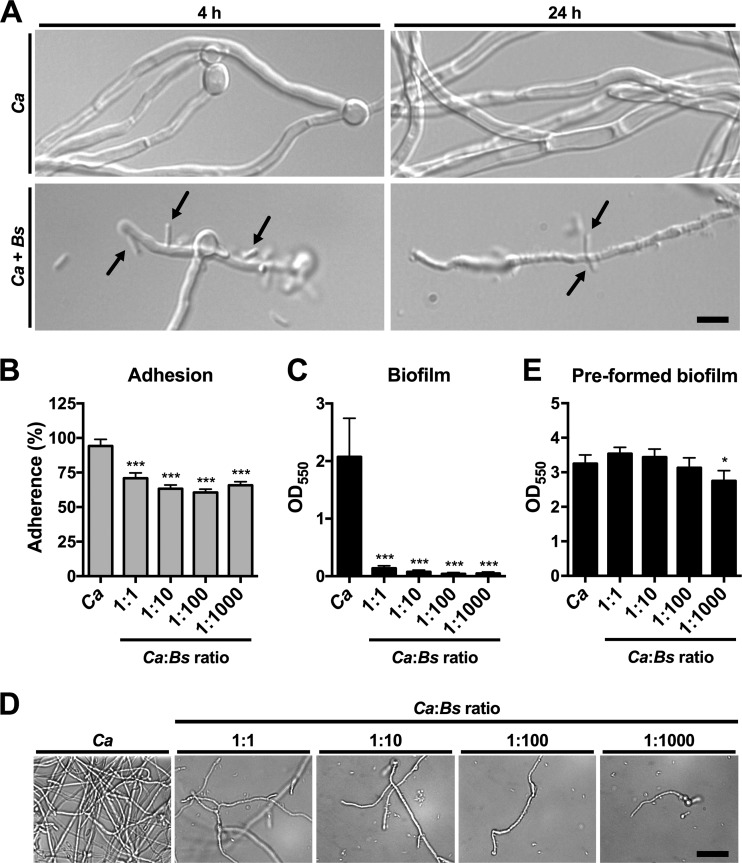
*B. safensis* attaches to *C. albicans* filaments, reduces fungal adhesion capacity, and prevents *de novo* biofilm formation. (A) DIC microscopy images of *C. albicans* grown for the indicated times under hypha-inducing conditions with or without bacteria. Arrows point to bacteria that have attached to fungal filaments. Note that after 24 h in the presence of *B. safensis*, *C. albicans* hyphae appear disintegrated and much thinner than those seen with the untreated control. Scale bar, 2 µm. (B) *B. safensis* reduces *C. albicans* adhesion to polystyrene in a dose-dependent manner. Fungal adherence is expressed relative to the initial inoculum. Results are the means + SEM of two independent experiments, each performed in quadruplicate. ***, *P* < 0.0001 (compared with the wild-type strain). (C) *B. safensis* inhibits *de novo C. albicans* biofilm formation in a dose-dependent manner. Fungal cells and bacterial cells were mixed at the indicated ratios and incubated under biofilm-inducing conditions. Biofilm formation was determined via crystal violet staining. Results are the means + SD of two independent experiments, each performed in quadruplicate. ***, *P* < 0.0001 (compared with the wild-type strain). (D) Microscopy images of *C. albicans* grown under biofilm-inducing conditions with or without bacteria (see panel C). Scale bar, 20 µm. (E) *B. safensis* does not reduce preformed *C. albicans* biofilms. Fungal biofilms were allowed to form and were then treated with the indicated ratios of bacteria. Note that a very modest reduction in fungal biofilm levels occurred only at the highest bacterial inoculum used in this experiment. Results are the means + SD of two independent experiments, each performed in quadruplicate. *, *P* < 0.01 (compared with the wild-type strain).

To gain further insight into the influence of *B. safensis* on *C. albicans* morphogenesis, we next analyzed fungus-bacterium interactions on the single-cell level. We discovered that bacterial cells appeared to attach to fungal hyphae as soon as 4 h following coinoculation into YPD medium (Fig. 5A). This effect was dependent on bacterial viability because heat-killed cells did not attach to fungal filaments (Fig. S7). After 24 h of coincubation, hyphal filaments appeared to be much thinner and partially disintegrated compared to fungus-only controls (Fig. 5A). We also performed analogous experiments using different ratios of fungal to bacterial cells and found that *C. albicans* hyphal length inversely correlated with the number of bacteria in the inoculum (Fig. S8). Furthermore, the higher the initial bacterial inoculum, the more bacteria were found to attach to *C. albicans* filaments (Fig. S8). Overall, these results confirmed that direct cell-cell contact appears to be a crucial aspect of the interaction between *B. safensis* and fungal pathogens and that the antifungus mechanism may be directed against the fungal cell surface.

10.1128/mBio.01537-17.7 FIG S7  Attachment of *B. safensis* to *C. albicans* hyphae requires live bacterial cells. Strains were grown in 10% FCS at 37°C and 5% CO_2_ for 24 h. White squares in the upper row panels indicate regions that are shown in a magnified view in the bottom row. Arrows point to bacterial cells attached to fungal hyphae, and triangles point to free-floating, unattached heat-killed (HK) bacterial cells. Scale bars indicate 10 µm (upper row) and 2 µm (lower row). The experiment was performed twice, and representative images are shown. Download FIG S7, TIF file, 6.2 MB. Copyright © 2017 Mayer and Kronstad. 2017 Mayer and Kronstad This content is distributed under the terms of the Creative Commons Attribution 4.0 International license.

10.1128/mBio.01537-17.8 FIG S8  *C. albicans* hypha length inversely correlates with bacterial inoculum. DIC microscopy images of *C. albicans*-*B. safensis* coincubations in 10% FCS at 37°C and 5% CO_2_ for 4 h are shown. Arrows point to bacterial cells attached to fungal filaments. Note that the higher the initial bacterial inoculum was, the higher the number of attached bacteria and the shorter the fungal hyphae. Scale bar, 10 µm. The experiment was performed twice, and representative images are shown. Download FIG S8, TIF file, 3.4 MB. Copyright © 2017 Mayer and Kronstad. 2017 Mayer and Kronstad This content is distributed under the terms of the Creative Commons Attribution 4.0 International license.

The yeast-to-hypha transition is a key process for the formation of *C. albicans* biofilms. Prior to biofilm formation, however, fungal cells need to adhere to a surface. The processes of adhesion and biofilm formation both have important clinical implications, as *C. albicans* biofilms often form on medical devices such as catheters and are extremely recalcitrant to antifungal therapy. In many cases, the only option is to surgically remove the catheters (27). We therefore next investigated whether *B. safensis* interfered with the adherence capacity and biofilm formation of *C. albicans*. Strikingly, the presence of bacteria inhibited adhesion of fungal cells to polystyrene (Fig. 5B) and drastically blocked *de novo* biofilm formation in a dose-dependent manner (Fig. 5C). Compared to the dense appearance of fungus-only biofilms, fungal cells exposed to bacteria were not able to form robust biofilms (Fig. 5D). Again, individual hyphal length was dependent on the dose of the initial bacterial inoculum. To evaluate the effects of *B. safensis* on *C. albicans* biofilms that had already formed, fungal biofilms were allowed to develop for 24 h, bacteria were added, and the biofilms were incubated for another 24 h. In this experiment, *B. safensis* did not have a major impact on *C. albicans* biofilm formation. A statistically significant but modest reduction in biofilm formation was detected only at the highest bacterial inoculum (Fig. 5E).

### Bacterial chitinase activity contributes to the interaction of *B. safensis* with *C. albicans*.

**FIG 6:**
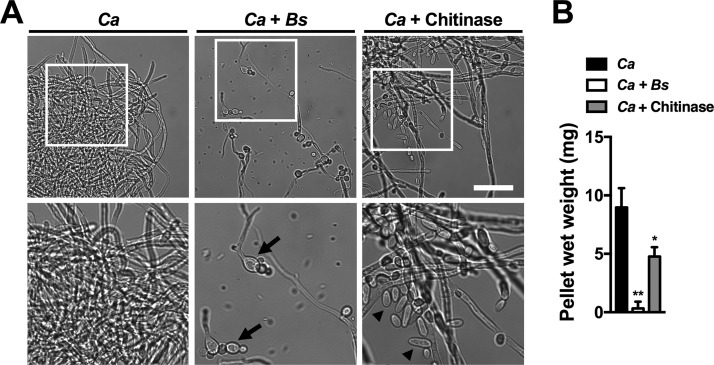
Exogenous chitinase partially inhibits *C. albicans* filamentation. (A) *C. albicans* was grown with or without *B. safensis* or 100 µg ml^−1^ chitinase in 10% FCS at 37°C and 5% CO_2_ for 24 h. White squares in the upper row panels indicate regions that are shown in a magnified view in the bottom row. Arrows point to abnormally shaped *C. albicans* cells, and arrowheads point to *C. albicans* yeast cells, which were not observed in the control sample without chitinase. Scale bar, 50 µm. (B) Semiquantitative evaluation of the impact of chitinase on *C. albicans* hypha formation. Results are the means + SD of three independent experiments. *, *P* < 0.05; **, *P* < 0.01.

On the basis of our finding that *B. safensis* chitinase activity is likely to contribute to inhibition of virulence factor production by *C. neoformans*, we next hypothesized that similar chitin-targeted enzymatic activity may be responsible for the suppression of *C. albicans* hypha formation by *B. safensis*. We therefore analyzed the effects of exogenous chitinase on *C. albicans* filamentation. Consistent with our hypothesis, chitinase significantly reduced hypha formation by *C. albicans*, though not as potently as *B. safensis* (Fig. 6). These results indicate that chitinase appears to contribute to the observed effects but that additional *B. safensis* factors are likely to be involved in this interspecies interaction.

**FIG 7:**
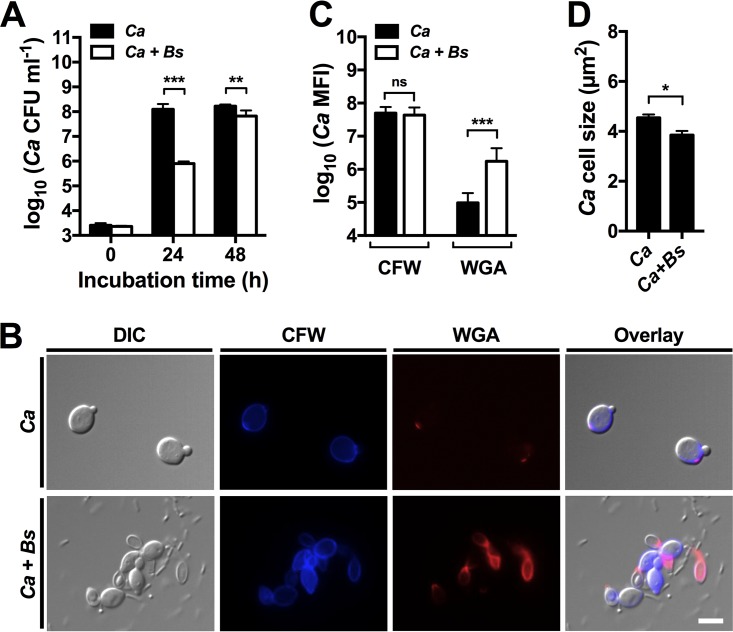
*B. safensis* delays *C. albicans* growth and targets the fungal cell wall. (A) Quantification of *C. albicans* growth in YPD with or without *B. safensis*. Results are the means + SD of three independent experiments, each performed in duplicate. **, *P* < 0.001; ***, *P* < 0.0001. (B) DIC and fluorescence microscopy images of *C. albicans* cells grown with or without *B. safensis* in YPD for 24 h and stained with CFW (calcofluor white; stains chitin) or with WGA (wheat germ agglutinin; stains chito-oligomers). Scale bar, 2 µm. (C) Quantification of CFW staining and WGA staining for the *C. albicans* cells described for panel B. MFI, mean fluorescence intensity. Results are the means + SD of 100 cells analyzed per condition. ns, not significant. ***, *P* < 0.0001. (D) Quantification of *C. albicans* cell size following growth in the absence or presence of *B. safensis*. Results are the means + SEM of 100 cells analyzed. *, *P* < 0.01.

To determine whether *B. safensis* affected general *C. albicans* growth, we performed additional bimicrobial interaction studies. *C. albicans* growth was reduced after 24 h of coincubation with the bacterium compared to the fungus-only control but reached similar viable cell numbers after 48 h (Fig. 7A). These results are similar to those obtained for *C. neoformans* (see Fig. 2B). Finally, we used the dye WGA to stain *C. albicans* cells following incubation with or without *B. safensis*. We detected significantly stronger WGA staining of fungal cells that had been exposed to bacteria than in the controls (Fig. 7B and C). Furthermore, the *C. albicans* cells were slightly reduced in size following dual-species interaction (Fig. 7D). This result indicates differences between *C. albicans* and *C. neoformans* in the morphological response of yeast cells to *B. safensis* (Fig. 7D and Fig. S5C). Overall, these results indicate that *B. safensis* likely targets the cell walls of *C. neoformans* and *C. albicans* through chitinase activity, resulting in destabilization of the cell wall architecture and ultimately leading to improper virulence factor expression.

## DISCUSSION

There are 10^7^ to 10^10^ prokaryotes per gram of soil in nature (28, 29). These enormous numbers make it highly likely that soil microbes, including bacteria, fungi, and protozoa, interact with each other, either directly or indirectly. Such interactions can have huge ecological, economic, and medical implications. Polymicrobial interaction studies are currently gaining momentum due to the realization that interspecies interactions strongly impact human health and disease (30–32). However, few studies have investigated the interactions among fungal pathogens naturally found in the environment and other niche-specific microbes. Specifically, whether environmental microbes impact fungal virulence traits is largely unknown. Here, we hypothesized that certain microorganisms in soil and on plants antagonistically interact with *C. neoformans* and *C. gattii*, two major human fungal pathogens found in these habitats. By screening 40 environmental microbes, we identified 10 bacteria of the genus *Bacillus* with activity against cryptococcal melanization.

**FIG 8:**
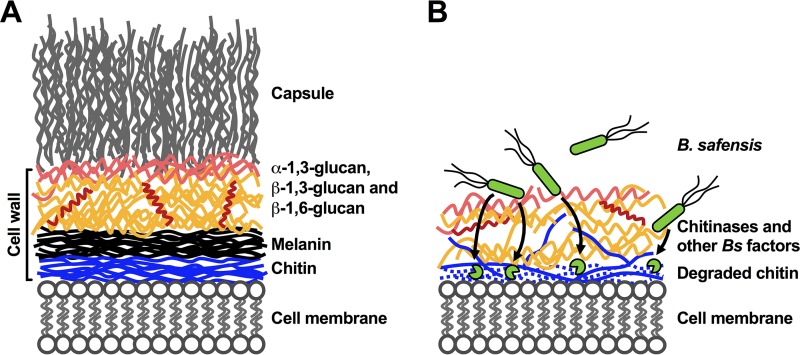
Proposed model depicting the impact of *B. safensis* on fungal cell walls. (A) Schematic view of the *C. neoformans* cell membrane and cell wall organization, with attached capsule polysaccharide. α-1,3-glucan is shown in light red, β-1,3-glucan is shown in orange, and β-1,6-glucan is shown in dark red. (B) Following direct cell contact, the environmental bacterium *B. safensis* produces chitinases and other factors which degrade fungal cell wall chitin and influence the interspecies interaction. The destabilization of the fungal cell wall architecture does not lead to fungal cell death but prevents proper anchoring of melanin and polysaccharide capsule components, thereby disarming the virulence factors of the fungal pathogen.

One bacterium, *B. safensis*, had a particularly strong capacity to block the melanization of *C. neoformans* and *C. gattii*. Anti-virulence factor activities against other fungal virulence traits, including capsule production and biofilm formation, were also identified. Furthermore, antifungal activity was not limited to basidiomycete fungi but was also detected against a major ascomycete fungal pathogen, *C. albicans*. *B. safensis* potently inhibited the morphological transition from yeast to hyphae and efficiently blocked *de novo* biofilm formation. Through genetic and phenotypic screens performed using a *C. neoformans* TF mutant library, we identified the fungal cell wall as a target of *B. safensis*. Furthermore, we provide evidence that *B. safensis* likely employs chitinases to destabilize the cell wall. These findings led us to propose a model (Fig. 8) in which *B. safensis* comes into close contact with fungal cells and secretes chitinases and other factors to degrade the chitin fibers that form the innermost layer of the cell wall (33). Chitin is a polymer of *N*-acetylglucosamine and is one of the most abundant biopolymers in nature. Because melanin is incorporated into the fungal cell wall via anchoring to the chitin layer in normal cells, chitinase-mediated disaggregation events may take away the foundation for proper melanin deposition (Fig. 8). As indicated, other factors may influence the cell wall as well as other potential targets in *C. neoformans*, including the plasma membrane.

Cryptococcal virulence factors such as laccases (enzymes mediating melanin biosynthesis) and capsule polysaccharides are delivered to the outer side of the cell via crossing the cell membrane and cell wall in vesicles (so-called “virulence delivery bags”) that transport virulence factor building blocks (5). Consistent with the idea of an impact of *B. safensis* on the cell membrane and fungal secretion dynamics, we observed aberrant uptake of the lipophilic dye FM4-64 into *B. safensis*-exposed *C. neoformans* cells compared to unexposed fungal cells. The model of cell wall destabilization via chitin degradation also provides a possible explanation for the observed reductions in *C. neoformans* capsule production during dual-species interaction (Fig. 8). Capsule polysaccharides are assembled onto the α-1,3-glucan layer of the cell wall, beneath which the β-1,3-glucan/β-1,6-glucan layer is anchored to chitin (5, 34). Mutants with impaired capsule formation are usually strongly reduced in virulence (12, 35, 36). Hence, our results reveal that *B. safensis* targets a fungal structure in a way that does not affect overall cell survival but is essential for proper virulence factor assembly.

Few studies have analyzed *C. neoformans*-bacterium dual-species interactions, and, to the best of our knowledge, the specific inhibition of cryptococcal virulence factor production has not been documented before. A pioneering study by Bulmer and colleagues described anticryptococcal activity exerted by two bacterial species isolated from pigeon guano, *Pseudomonas aeruginosa* and *Bacillus subtilis* (37). Another landmark publication by Casadevall and colleagues established a potential role of soil amoeba in shaping the virulence repertoire of *C. neoformans* during dual-species interactions in the environment (38). Amoebae show remarkable similarities with macrophages, key cells of the human immune system, and it was found that fungal capsule and melanin production protected *C. neoformans* following ingestion by amoebae (38). More recently, the soil bacterium *Acinetobacter baumannii* was found to inhibit *C. neoformans* growth in a fungal serotype-dependent manner. In contrast to our findings obtained with *B. safensis*, however, *A. baumannii* induced *C. neoformans* capsule enlargement (39).

It is remarkable that our isolation procedure appears to have selected for bacteria of the genus *Bacillus*. We used YPD and LB media for isolation, and it is possible that these nutrient-rich, complex media specifically promote growth of this environmental bacterial genus. Indeed, several studies in which bacteria were isolated from environmental soil or plant samples identified *Bacillus* as the predominant bacterial genus (40, 41). The antimelanization effect, however, was not general with respect to the *Bacillus* genus, because some bacteria belonging to this genus did not display antifungal activity.

*B. safensis* is a Gram-positive, motile, spore-forming, ubiquitous soil bacterium that was first isolated from the spacecraft assembly facility at NASA’s Jet Propulsion Laboratory in Pasadena, CA, USA (42). *B. safensis* is a close relative of *Bacillus pumilus*, a soil bacterium that was found also to cause contamination of spacecraft equipment (43). Spores of *B. pumilus* are extremely resistant to oxidative and UV stress (44). Interestingly, several studies have demonstrated antifungal chitinase production by *B. pumilus*, and this bacterium is used as a commercial probiotic and as a biopesticide in agriculture (45–48). Moreover, bacteria of the genus *Bacillus* in general appear to possess particularly potent antifungal activity. A mixture of five bacterial species, three of which were *Bacillus cereus*, *Bacillus megaterium*, and *Bacillus mojavensis*, for example, was found to protect tobacco (*Nicotiana attenuata*) from *Fusarium* and *Alternaria* infection (49).

We used *C. gattii*-positive soil samples from Vancouver Island to increase the likelihood of identifying microbes with specific activity against cryptococcal cells (15). Vancouver Island is a hot spot for *C. gattii* and has been the site of an outbreak of cryptococcosis in recent years (50, 51). Importantly, *B. safensis* also displayed potent antimelanization activity against *C. gattii*. It remains to be determined whether soils from areas without *C. gattii* or *C. neoformans* harbor similar microbial repertoires. As a first analysis, we focused only on easily cultivable microbes. We are aware that one limitation of our study is the fact that we did not isolate “uncultivable” environmental microbes that may exert important antifungal activities. Future studies could employ strategies such as the use of an iChip to isolate and culture uncultivable microbes from soil (52).

We detected moderate melanin shedding activity for *B. safensis* monocultures in liquid l-DOPA, indicating that these bacteria may produce an enzyme capable of metabolizing l-DOPA. Indeed, *B. safensis* produces at least one laccase (16). However, we argue that this activity does not interfere with the effect on cryptococcal melanization because (i) *B. safensis*-spent medium provides enough l-DOPA for *C. neoformans* to melanize; (ii) the measured values of bacterial melanin shedding were comparably small; and (iii) the bacterial colonies or cell periphery did not turn black, which would have been indicative of laccase-mediated pigment production.

We determined that bacteria required close contact with fungal cells to exert their antifungal effects. This was supported by several findings. First, heat-killed *B. safensis* cells did not block cryptococcal melanization. Second, *B. safensis* attached directly to *C. albicans* filaments. Finally, bacterial cells were found to swarm around fungal colonies on solid agar plates. Swarming in *Bacillus* is mediated by flagella and secretion of surfactins, lipopeptides that reduce tension between the substrate and the bacterial cells to allow gliding over surfaces (53, 54). What the exact fungal cues are that direct bacterial chemotaxis is unknown, but fungal polysaccharide shedding may contribute to this phenomenon. The requirement of direct cell-cell contact for bacterial antagonism has been described before for the interaction between *C. neoformans* and *Staphylococcus aureus*, an important bacterial pathogen of humans. *S. aureus* attached to and killed *C. neoformans* in a process that was dependent on presence of fungal capsule polysaccharide (55). Furthermore, contact-dependent growth inhibition events have been described for *P. aeruginosa*-*C. neoformans* dual-species interactions. Bacteria inhibited *C. neoformans* via production of the quorum-sensing molecule pyocyanin (56). These studies indicate that direct bacterium-fungus interactions may be common but that the individual mechanisms of interaction can differ significantly.

*B. safensis* blocked *C. neoformans* capsule formation, and this prompted us to investigate fungal biofilm formation since the capsule matrix surrounding fungal biofilms is composed of the same glucuronoxylomannan (GXM) molecules that are the main building blocks of the polysaccharide capsule (25). Consistent with our hypothesis, *C. neoformans* biofilm formation was significantly inhibited by *B. safensis*. The values for biofilms formed by the fungus-bacterium cocultures are likely to be overestimates since this analysis did not discriminate between the two organisms and *B. safensis* formed considerable biofilms on its own.

*C. albicans* is a major opportunistic human fungal pathogen and can cause infections in immunocompromised persons that range from superficial infections of the skin and mucosal surfaces to life-threatening systemic infections with high mortality rates (14). A key virulence factor in this fungus is the capacity to transition between the yeast and hyphal forms, both of which are required for infection (26). Yeast cells can adhere to biotic surfaces (e.g., oral epithelial cells) and abiotic surfaces (e.g., catheters and polystyrene) and are believed to represent the morphology that mediates dissemination through the bloodstream. The hyphal form has been shown to mediate tissue penetration, both through active fungus-driven mechanical forces and by induced endocytosis by host cells (57–59). Hence, both candidal cell morphologies play important roles during pathogenesis. *B. safensis* significantly reduced *C. albicans* adhesion to polystyrene and strongly inhibited hypha formation. Because adhesion and hypha formation are prerequisites for biofilm formation in this pathogen, we hypothesized that *B. safensis* may have antibiofilm activities. Indeed, *B. safensis* potently blocked *de novo C. albicans* biofilm formation, while preformed *C. albicans* biofilms were resistant to *B. safensis* treatment. This is likely due to the fact that fungal cell walls are dynamically modified during growth, division, and morphogenesis. Indeed, fungi encode chitinases that have important roles in cell wall plasticity (60). These fungal chitinase activities, however, must be tightly regulated to prevent autodirected cell wall damage. Preformed *C. albicans* biofilm cells are encased in an extracellular matrix that probably protects these cells from *B. safensis* chitinase activity.

Several studies have investigated the effects of bacteria on *C. albicans* hypha formation (61–64). Perhaps the best-studied interactions are those of *C. albicans* and *P. aeruginosa*, an important bacterial pathogen of humans (62, 65–67). Similarly to our findings with *B. safensis*, *P. aeruginosa* was demonstrated to attach to fungal filaments, and bacteria specifically killed fungal hyphae via mechanisms dependent on pili, phospholipase C, and phenazine secretion (62). Furthermore, a recent study demonstrated a role for the *Enterococcus faecalis* toxin EntV in blocking *C. albicans* filamentation, biofilm production, and virulence in a murine model of oropharyngeal candidasis (68). These studies indicate that certain bacteria interact with *C. albicans* and appear to preferentially target the hyphal morphology.

Several lines of evidence pointed toward a role of chitinase activity in mediating anti-virulence factor activities during fungus-bacterium dual-species interaction. First, we identified several *C. neoformans* cell surface-defective TF mutants that displayed altered interaction upon exposure to *B. safensis* compared with the *C. neoformans* wild type. Specifically, the *nrg1*Δ, *sre1*Δ, and *ert1*Δ mutants all have cell membrane and/or cell wall defects (18, 19, 69). Second, the cell membrane-stabilizing agent sorbitol rescued *B. safensis*-mediated inhibition of melanization, and this effect was not observed with the *nrg1*Δ mutant. Third, previously published data revealed a striking dysregulation of the chitin-synthesis machinery in *nrg1*Δ cells (19), and exposure to *B. safensis* resulted in *nrg1*Δ cell morphologies reminiscent of those of chitin synthase mutants (22, 23). Fourth, coincubation with *B. safensis* and the chitin-inhibiting agent calcofluor white resulted in synergistic inhibition of *C. neoformans* growth. Fifth, the chitinase inhibitor BisC partially rescued *C. neoformans* melanization during coculture with *B. safensis*. And sixth, WGA staining revealed significantly more chito-oligomers on the cell surfaces of *C. neoformans* and *C. albicans* cells following exposure to *B. safensis*. Conceivably, *B. safensis* may also produce glucanases in addition to chitnases; however, we did not detect synergism of inhibition of *C. neoformans* growth between *B. safensis* and Congo red, a dye that inhibits β-1,3-glucan. Consistent with our results on antifungal chitinase potency, a recent study from Yoo and Choi demonstrated anti-*C. albicans* and anti-*Cryptococcus* activity of a chitinase isolated from the mushroom *Coprinellus congregatus* (70).

Despite the similarities between the effects on *C. neoformans* and *C. albicans*, we also noted an interesting, medium-dependent difference during bimicrobial interactions with *B. safensis*. While *C. albicans* cells were moderately reduced in size following exposure to bacteria compared to fungal monocultures, *C. neoformans* cells nearly doubled in size. This increase in cell size was observed in nutrient-rich YPD medium (Fig. S5C) but not in capsule-inducing medium (Fig. 4B) and may have been related to titan cell formation by *C. neoformans*, a process occurring during *in vivo* infection and associated with increased tolerance of attack from immune cells (71, 72). Cell enlargement upon contact with *B. safensis* may therefore be a fungal response aimed at self-protection from bacterial attack under these culture conditions.

Due to the absence of cell walls in human cells, the fungal cell wall represents an excellent target for the development of antifungal drugs. Indeed, current antifungal development programs specifically aim to target this pathogen structure. Nikkomycin Z, for example, is a compound that targets fungal cell wall chitin synthesis and is currently in clinical trial for the treatment of coccidioidomycosis (73, 74). Humans produce chitinases, and it is hypothesized that these enzymes have antimicrobial functions (75). Initial studies have shown that administration of chitotriosidase, the recombinant human chitinase, to mice reduced cryptococcal and candidal infection (76, 77). Moreover, it was recently shown that chitotriosidase recognizes cryptococcal chitin during infection and promotes pathologic type-2 helper T cell responses (78).

In summary, we have identified an environmental soil bacterium that specifically blocks virulence factor elaboration by human-pathogenic fungi without affecting overall fungal growth. We identified bacterial chitinase activity as a major contributing factor that mediates these effects and propose that identifying additional microbes with pathogenicity-specific activities may represent a promising approach to identify novel, pathogen-specific drug targets.

## MATERIALS AND METHODS

### Strains and growth conditions.

*C. neoformans* var. *grubii* strain H99 (serotype A), *C. gattii* strain R265 (molecular type VGIIa; serotype B), and *C. albicans* strain SC5314 were used as wild-type controls. Other strains used in this study are listed in Tables S1 and S2. Fungal strains were routinely maintained on YPD agar (1% yeast extract, 2% Bacto-peptone, 2% d-glucose, 2% agar). Overnight cultures were grown in liquid YPD medium in a shaking incubator at 30°C and 180 rpm. The environmental microbes (see Table S1) and *E. coli* strain DH5α were cultivated on LB agar (1% Bacto-tryptone, 0.5% yeast extract, 1% NaCl, 2% agar), and overnight cultures were grown in liquid LB in a shaking incubator at 30°C and 180 rpm.

10.1128/mBio.01537-17.10 TABLE S2  Fungal and bacterial strains used in this study. Download TABLE S2, DOCX file, 0.1 MB. Copyright © 2017 Mayer and Kronstad. 2017 Mayer and Kronstad This content is distributed under the terms of the Creative Commons Attribution 4.0 International license.

### Isolation of environmental microbes.

Environmental microorganisms were isolated from *C. gattii*-positive soil samples (from Vancouver Island, BC, Canada) (15) and from plant leaves of *Aucuba japonica* and *Urtica dioica* (from the University of British Columbia [UBC] campus, BC, Canada). For each soil sample, ~50 mg was suspended in sterile phosphate-buffered saline (PBS), vigorously shaken, and incubated statically at room temperature (RT) for 20 min to allow undissolved particles to sink to the bottom of the tube. Next, 100 µl of supernatant was plated onto LB agar and incubated at 30°C for 24 to 48 h. Single colonies were then restreaked twice to ensure enrichment of single organisms. For isolation of microbes from plants, upper leaf surfaces were gently pressed onto YPD agar for 5 to 10 s, and plates were incubated at 30°C for 24 to 48 h. Colonies were restreaked three times onto YPD. Microorganisms were kept in 25% glycerol at −80°C for long-term storage.

### Identification of microbes.

Selected microbes were identified at the genus level by 16S rRNA sequencing according to previously published protocols (79), with minor modifications. Briefly, genomic DNA (gDNA) was isolated by bead beating and phenol-chloroform extraction according to standard protocols. Universal primers 8F (5′-AGAGTTTGATCCTGGCTCAG-3′) and 1492R (5′-GGTTACCTTGTTACGACTT-3′) were then used to amplify the 16S rRNA gene sequence. PCR experiments were performed in a total volume of 50 µl and contained 32.5 µl water, 10 µl buffer HF (5×), 4 µl deoxynucleoside triphosphate (dNTP) mix (with a 2.5 mM concentration of each dNTP), 1 µl forward primer (8F), 1 µl reverse primer (1492R), 1 µl gDNA (30 ng µl^−1^), and 0.5 µl Phusion DNA polymerase (New England Biolabs). The following PCR cycle was used for amplification: initial activation at 98°C for 30 s; 32 cycles at 98°C for 10 s, 56°C for 30 s, and 72°C for 45 s; and a final extension at 72°C for 10 min. PCR products were purified using a PCR purification kit (Fermentas) and sequenced using primer 8F and/or primer 1492R (Genewiz). Nucleotide sequences were then assessed for base-caller errors, and ~700-bp gene sequences were compared with reference 16S rRNA gene sequences by BLAST analysis at the National Center for Biotechnology Information (NCBI) website (https://blast.ncbi.nlm.nih.gov/Blast.cgi). The 16S rRNA gene sequences were deposited in GenBank (see below).

### Growth curves and CFU assays.

A potential impact of interspecies interactions on fungal growth was evaluated by cell number determinations performed using a hemocytometer, optical density measurements, or CFU analyses.

The analysis of cryptococcal growth dynamics based on cell number was performed according to previously published reports (80), with minor modifications. Briefly, fungal and bacterial overnight cultures were washed twice in PBS and resuspended in PBS. Fungal cells alone (2 × 10^3^ cells ml^−1^) or in a mix of fungal cells (2 × 10^3^ cells ml^−1^) and bacterial cells (2 × 10^5^ cells ml^−1^) were then incubated in 5 ml l-DOPA or YPD in the dark at 30°C and 180 rpm. At the indicated time points, culture aliquots were diluted as required and fungal cell numbers determined using a hemocytometer. Note that fungal cells were readily differentiable from bacterial cells in the dual-species culture due to their significantly greater size and distinct morphology.

Growth curve analyses were performed for *C. neoformans* and *B. safensis* monocultures in sterile, 96-well microtiter plates. Overnight fungal or bacterial cultures were washed twice in PBS and adjusted to an OD_600_ of 0.1 in YPD or in YPD plus 50 µg ml^−1^ gentamicin. The total medium volume per well was 200 µl. The plates were sealed with a sterile adhesive foil and incubated in a microplate reader (Infinite M200; Tecan) at 30°C. Growth of the strains was then recorded by measuring the OD_600_ at 30-min intervals for up to 72 h.

For CFU analyses, bacterial and fungal overnight cultures were washed twice in PBS and resuspended in PBS. Fungal cells alone (10^3^ cells ml^−1^) or in a mix of fungal cells (10^3^ cells ml^−1^) and bacterial cells (10^6^ cells ml^−1^) were incubated in 1 ml YPD, YPD plus 200 µg ml^−1^ Congo red (CR), or YPD plus 400 µg ml^−1^ CFW at 30°C and 180 rpm. At the indicated time points, 50 µl and 100 µl of the appropriate dilutions were plated onto YPD plus 30 µg ml^−1^ gentamicin. Plates were incubated for 2 to 3 days at 30°C and fungal colonies counted.

### Melanin production.

Melanin production was examined on solid or in liquid l-3,4-dihydroxyphenylalanine (L-DOPA; Sigma) medium containing 0.1% glucose. To screen for microbes with antimelanization activity, overnight bacterial and cryptococcal cultures were mixed at a 1:1 ratio and 7 µl was dropped onto l-DOPA agar. Bacterium- and fungus-only controls were included. Plates were incubated in the dark at 30°C or 37°C for 48 h before being photographed. For some experiments, bacterial or fungal overnight cultures were washed twice in PBS and adjusted to an OD_600_ of 20 or 4, respectively, before being mixed and dropped onto l-DOPA agar. Washing with PBS did not affect the bacterial antimelanization activity. To analyze the effects of chitinase on formation of melanin, fungal cells were resuspended in 1 mg ml^−1^ chitinase (from *Streptomyces griseus*; Sigma) solution before being spotted onto l-DOPA agar. To investigate if *B. safensis* viability is required for the antimelanization activity, bacterial cells were grown overnight, heat killed (15 min at 95°C), and exposed to *C. neoformans* on l-DOPA agar at 30°C for 48 h.

The effects of membrane stabilization or chitinase inhibition on *B. safensis*-mediated inhibition of *C. neoformans* melanization were tested by incubating monospecies or dual-species cultures on l-DOPA agar containing 1.5 M sorbitol or 2.5 mM bisdionine C (BisC), respectively.

To analyze the impact of *B. safensis* supernatant on melanin production by *C. neoformans*, bacteria were grown for 48 h at 30°C and 180 rpm in 50 ml YPD or potato dextrose broth (PDB; Difco) or yeast nitrogen base–low-iron medium (YNB-LIM [81]). Cultures were then centrifuged for 30 min at 4,200 rpm, and ~10 ml of supernatant was subjected to filter sterilization (0.2 µm pore size) and concentrated using 10 kDa-molecular-weight-cutoff tubes (Amicon Ultra) following the manufacturer’s instructions. Concentrated supernatant from each bacterial preculture condition was then mixed with an overnight culture of *C. neoformans* at a ratio of 1:1 (vol/vol) and incubated on l-DOPA agar at 30°C for 48 h.

To analyze if *B. safensis*-spent l-DOPA medium provides enough substrate for *C. neoformans* to still melanize, bacterial cells were grown in l-DOPA at 30°C for 48 h and the supernatant was subjected to filter sterilization (0.2 µm pore size) and reinoculated with *C. neoformans*. Uninoculated spent medium was included as a control. Samples were incubated at 30°C and 180 rpm for 48 h before being photographed.

Melanin shedding was analyzed according to previously published protocols (82), with minor modifications. Briefly, overnight fungal or bacterial cultures were washed twice in PBS and adjusted to 10^6^ cells ml^−1^ or 10^9^ cells ml^−1^ in PBS, respectively. The same volumes of fungal and bacterial cells were mixed (final ratio, 1:1,000), and 100 µl was added to 5 ml l-DOPA. Fungus-only and medium-only controls were included, and samples were incubated in the dark at 30°C and 180 rpm for 46 h. Next, 1 ml culture was centrifuged at 1,000 × *g* for 10 min, 100 µl of supernatant was transferred to a 96-well plate, and the OD_405_ was measured in a microplate reader (Infinite M200; Tecan).

### Capsule formation.

To investigate *C. neoformans* capsule formation in the presence or absence of *B. safensis*, stationary-phase fungal or bacterial cultures were washed once in PBS and resuspended in PBS. For capsule induction, 70 µl of fungal cells alone or bacterial cells alone or of a mixture of fungal cells and bacterial cells was added to 2 ml 10% fetal calf serum (FCS; Gibco) or to 2 ml low-iron capsule induction medium (CIM; 5 g liter^−1^ glucose, 5 g liter^−1^ l-asparagine, 0.4 g liter^−1^ K_2_HPO_4_, 0.25 g liter^−1^ CaCl_2 ⋅ _2H_2_O, 0.08 g liter^−1^ MgSO_4 ⋅ _7H_2_O, 4.78 g liter^−1^ HEPES, 1.85 g liter^−1^ NaHCO_3_ [dissolved in Chelex 100 resin-treated water], pH 7.4) supplemented with 100 µg ml^−1^ chitinase or left unsupplemented and was incubated at 30°C and 180 rpm for 48 h. Samples were then stained with India ink to enable visualization of the polysaccharide capsule by differential interference contrast (DIC) microscopy.

### TF mutant library screen.

To identify a potential mechanism for the observed suppression of cryptococcal melanization by *B. safensis*, we performed a genomic screen using a *C. neoformans* transcription factor (TF) mutant library (18). The TF library encompasses 322 mutants with deletions in 155 different TFs (with at least two independent deletion strains per TF). In this study, we screened two independent mutants each for 154 of these TFs (a total of 308 mutants). TF mutants were grown overnight in 96-well plates in YPD at 30°C. TF mutant cultures were then diluted 1:40 in liquid l-DOPA and incubated either alone or with bacterial cells (final OD_600_ = 0.0025) in a final volume of 200 µl l-DOPA. Plates were incubated in the dark at 30°C for 5 days. To assess overall growth, the OD_405_ was then measured for each plate using a microplate reader (Infinite M200; Tecan).

### Hypha formation.

*C. albicans* hypha formation in the presence or absence of bacteria was investigated both on solid and in liquid media. For solid plate assays, overnight *C. albicans*, *B. safensis*, and *E. coli* cultures were washed twice in PBS and adjusted to 2 × 10^6^ cells ml^−1^ each in PBS. Fungal and bacterial cells were mixed in a 1:1 ratio, and 7 µl was spotted onto solid water agar supplemented with 10% FCS. Fungus-only cultures were included as a positive control. Plates were incubated at 30°C for 8 to 9 days before being photographed.

For analysis of filament formation in liquid medium, overnight fungal and bacterial cultures were washed twice in PBS and cell numbers were adjusted to 10^7^ cells ml^−1^ and 10^8^ cells ml^−1^ in PBS, respectively. A 10-µl volume of fungal cells ± 10 µl of bacterial cells was then added to 5 ml YPD or to 2 to 5 ml 10% FCS supplemented with or without 100 µg ml^−1^ chitinase. YPD cultures were incubated at 30°C and 180 rpm for 24 h to induce yeast-phase growth, and cultures prepared in 10% FCS were incubated at 37°C and 180 rpm for 24 h to induce hypha formation. Samples were then analyzed by DIC microscopy. To estimate *C. albicans* yeast and hyphal mass production, samples were kept at 4°C overnight to allow the heavier fungal cells to sink to the bottom of the glass tube. Next, the supernatant, containing mostly bacterial cells, was carefully removed and the remaining cells were resuspended in PBS and transferred to preweighed microcentrifuge tubes. The supernatant was carefully removed in two centrifugation steps (3 min at 15,000 rpm), and the microcentrifuge tubes containing the pellets were weighed. Note that this procedure provides only an estimate of fungal yeast and hypha production, as some bacterial cells are likely to be carried over following the overnight static incubation step. Due to the small size of bacterial cells relative to fungal cells, however, these values should be negligible.

To study *C. albicans* hypha formation with or without *B. safensis* on the single-cell level, overnight fungal and bacterial cultures were washed once in PBS and resuspended to levels of 10^7^ cells ml^−1^ and 10^9^ cells ml^−1^ in PBS, respectively. For coculture, 10 µl fungal cells and 10 µl bacterial cells (ratio of 1:100) were added to 3 ml 10% FCS and incubated in a humidified incubator at 37°C with a 5% CO_2_ atmosphere. Fungus-only cultures were used as controls. After 4 h and 24 h, aliquots were taken and analyzed by DIC microscopy.

To investigate the impact of various fungus-bacterium ratios on *C. albicans* filamentation, hypha induction assays were performed in sterile, polystyrene, flat-bottom, 24-well microtiter plates (Corning). Overnight fungal or bacterial cultures were washed three times in PBS and cell numbers determined using a hemocytometer. *C. albicans* was adjusted to 2 × 10^4^ cells ml^−1^ in 10% FCS, and 500 µl was added per well (*C. albicans*-only control). For fungus-bacterium cocultures, the same number of fungal cells (10^4^ cells per well) was exposed to bacterial cells at ratios of 1:1, 1:10, 1:100, and 1:1,000 in a total volume of 500 µl 10% FCS per well. Plates were then incubated for 4 h in a humidified incubator at 37°C with a 5% CO_2_ atmosphere. Next, all wells were washed three times with PBS. The surface-adherent fungal cells with or without attached bacteria were fixed with 4% formaldehyde, and the plates were kept at 4°C for short-term storage until microscopic analysis.

To study the effect of *B. safensis* viability on the capacity to attach to *C. albicans* hyphae, fungal cells were exposed to live or heat-killed (15 min, 98°C) bacterial cells in 10% FCS at 37°C and 5% CO_2_ for 24 h. Aliquots were then analyzed by fluorescence microscopy.

### Adhesion assays.

*C. albicans* adhesion assays with or without *B. safensis* were performed in sterile, polystyrene, flat-bottom, 24-well microtiter plates (Corning). Overnight fungal or bacterial cultures were washed three times in PBS and cell numbers determined. *C. albicans* was adjusted to 2 × 10^4^ cells ml^−1^ in 10% FCS, and 500 µl was added per well (*C. albicans*-only control). For fungus-bacterium cocultures, the same number of fungal cells (10^4^ cells per well) was exposed to bacterial cells at ratios of 1:1, 1:10, 1:100, and 1:1,000 in a total volume of 500 µl 10% FCS per well. Plates were then incubated for 45 min in a humidified incubator at 37°C with 5% a CO_2_ atmosphere. All wells were washed three times with PBS to remove bacteria and nonadherent *C. albicans* cells. The surface-adherent fungal cells were fixed with 4% formaldehyde, and plates were kept at 4°C for short-term storage until microscopic analysis. Quantification of *C. albicans* adherence was performed using an inverse microscope (Zeiss ID03). The number of adhered cells was determined by counting cells in 20 random high-power fields (HPFs) (size, ~0.238 mm^2^ per well). The experiment was performed twice in quadruplicate (160 HPFs in total examined per condition).

### Biofilm assays.

*C. neoformans* biofilm experiments were performed with or without *B. safensis* by crystal violet staining as previously described (83), with minor modifications. Briefly, overnight fungal and bacterial cultures were washed three times in PBS and resuspended in Dulbecco’s modified Eagle media (DMEM; Gibco) to reach a final concentration of 10^7^ cells ml^−1^ as individual bacterial or fungal cultures or as a mixture of the two organisms (1:1 ratio, with 10^7^ cells ml^−1^ each). Next, 300 µl of fungal cells, bacterial cells, or mixed fungal cells-bacterial cells were transferred into individual wells of sterile, polystyrene, flat-bottom, 24-well microtiter plates (Corning). Wells with DMEM only were included as controls. Plates were incubated at 30°C or at 37°C for 48 h in plastic bags (to avoid medium evaporation). Each well was then washed twice with sterile water and air-dried for 5 min at RT. For biofilm quantification, 100 µl 0.3% crystal violet solution was added to each well (including the medium-only control wells) and plates were incubated at RT for 5 min. Next, each well was thoroughly washed twice with sterile water and biofilms were destained with 200 µl 100% ethanol for 5 min at RT. Finally, 75 µl of destaining solution was transferred to a new 96-well microtiter plate and the OD_550_ measured in a microplate reader (Infinite M200; Tecan). Medium-only control values were subtracted from all measurements.

For analysis of *de novo C. albicans* biofilm formation with or without *B. safensis*, overnight fungal and bacterial cultures were washed three times in PBS and resuspended in 10% FCS to a final concentration of 2 × 10^4^ cells ml^−1^. Next, 500 µl of cells was transferred to wells of sterile, polystyrene, flat-bottom, 24-well plates (Corning). For fungus-bacterium cocultures, equivalent numbers of fungal cells (10^4^ cells per well) were exposed to bacterial cells at ratios of 1:1, 1:10, 1:100, and 1:1,000 in a total volume of 500 µl 10% FCS per well. Wells with 10% FCS medium only were included as controls. Plates were then incubated for 24 h in a humidified incubator at 37°C with a 5% CO_2_ atmosphere. Next, wells were washed three times with PBS and air-dried for 5 min at RT. Biofilm quantification was then performed using crystal violet staining as described above for *C. neoformans*.

To investigate the effect of *B. safensis* on preformed *C. albicans* biofilms, fungal cells were allowed to form biofilms for 24 h according to the protocol described above. Supernatants were then removed, and 10^4^, 10^5^, 10^6^, or 10^7^ bacterial cells in 10% FCS (corresponding to a fungus-bacterium ratio of 1:1, 1:10, 1:100, or 1:1,000 with respect to initial inocula) were added to fungal biofilm-containing wells. Plates were then returned to 37°C and 5% CO_2_ and further incubated for 24 h. Next, each well was washed three times with PBS and biofilm production quantified via crystal violet staining as described above.

### Cell membrane and cell wall staining.

To assess the impact of *B. safensis* on the *C. neoformans* cell membrane, monospecies or dual-species cultures were grown overnight in YPD at 30°C and 180 rpm and cells were washed in PBS and stained with FM4-64 (Invitrogen) at a final concentration of 20 µM in the dark at RT for 20 min (84). Cells were then washed twice in PBS and analyzed by fluorescence microscopy. For analysis of a potential impact of bacteria on fungal cell wall chitin, monospecies or dual-species cultures were grown overnight in YPD at 30°C and 180 rpm, washed three times in PBS, and fixed in 4% formaldehyde at 4°C for 30 min. Next, cells were stained with calcofluor white (CFW; Sigma) at a final concentration of 25 µM in the dark at 37°C for 30 min. Cells were then washed three times in PBS and analyzed by fluorescence microscopy. For simultaneous detection of chito-oligomers, cultures were prepared and washed in PBS as described above and then stained with fluorescein-conjugated wheat germ agglutinin (WGA; Invitrogen) at a final concentration of 5 µg ml^−1^ in the dark at 37°C for 30 min. Cells were then washed three times in PBS, stained with CFW as described above, and visualized by fluorescence microscopy using appropriate filter sets.

### Microscopy.

*C. albicans* adhesion capacity and fungal-bacterial biofilm formation were analyzed using inverted microscopes (Zeiss ID03, Olympus CKX41, and Olympus IX81). CFW, WGA, and FM4-64 staining results were investigated by fluorescence microscopy (Zeiss Axioplan 2) using appropriate filter sets. Micrographs were captured (Photometrics Cool Snap HQ^2^ camera) and analyzed with the relevant software program (Molecular Devices MetaMorph or 3i Slidebook). Quantifications of CFW and WGA mean fluorescence intensities (MFI) and cell size measurements were performed using Fiji (85).

### Statistics.

Data were visualized and statistically analyzed using GraphPad Prism version 7.0 (GraphPad Software, Inc., USA). Statistical tests were performed by one-way analysis of variance (ANOVA) followed by a Bonferroni correction (melanin shedding assays, adhesion assays, biofilm assays), by two-way ANOVA followed by a Bonferroni correction (CFU data, *C. albicans* pellet wet weights), or by Student’s *t* test (capsule size, CFW and WGA staining, fungal cell size, *B. safensis* growth in the presence of sorbitol). *P* values of ≤0.05 were considered to be significant.

### Accession number(s).

The 16S rRNA gene sequences determined in this work were deposited in GenBank (accession numbers MF347931 to MF347938).
