# Supplementary figures and images for: Disarming Fungal Pathogens: Bacillus safensis Inhibits Virulence Factor Production and Biofilm Formation by Cryptococcus neoformans and Candida albicans
Source: mBio. 2017 Oct 3;8(5):e01537-17. doi: 10.1128/mBio.01537-17 (PMC5626971; doi:10.1128/mBio.01537-17)

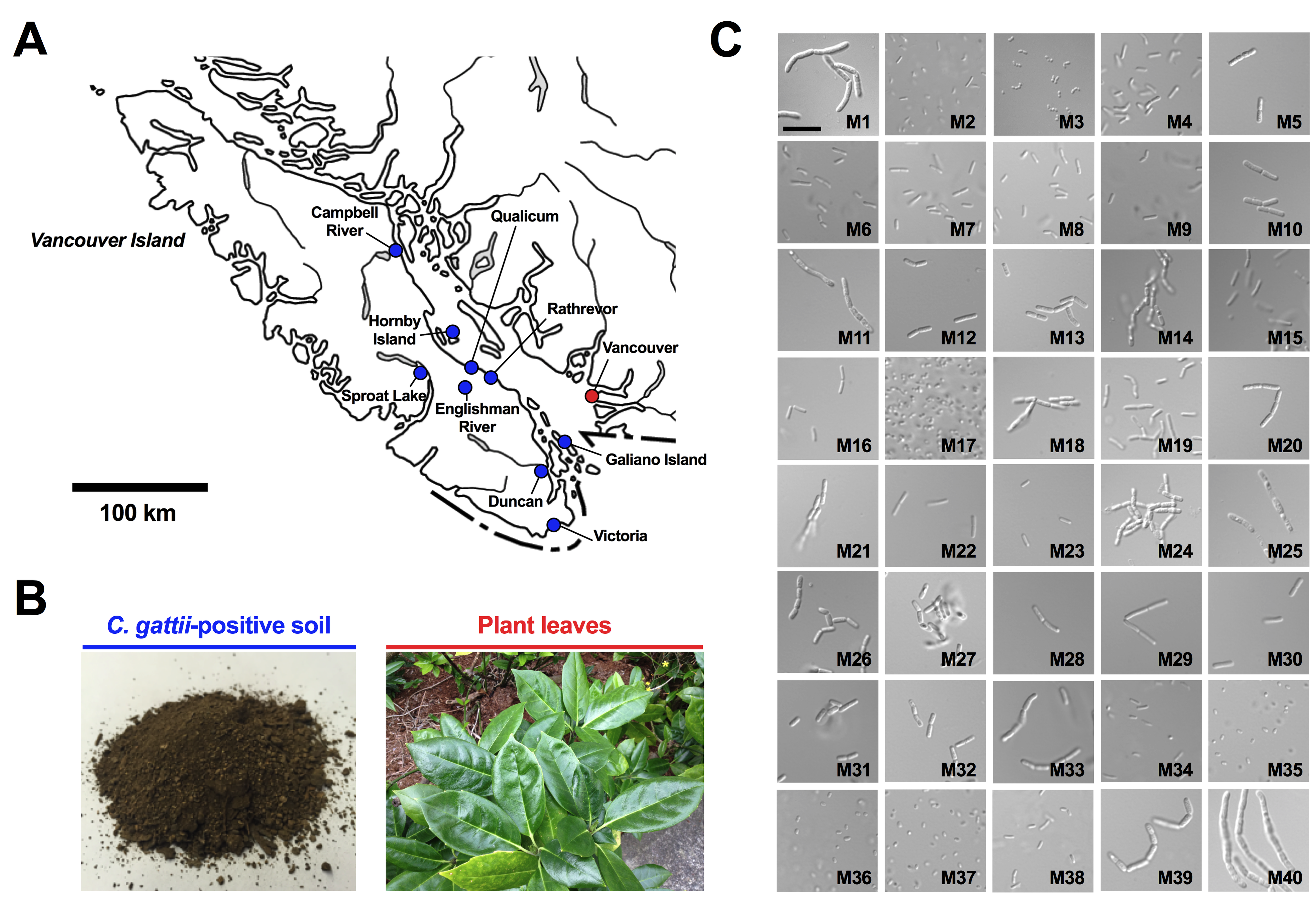

Supplement: FIG S1 [file mbo005173512sf1.tif]

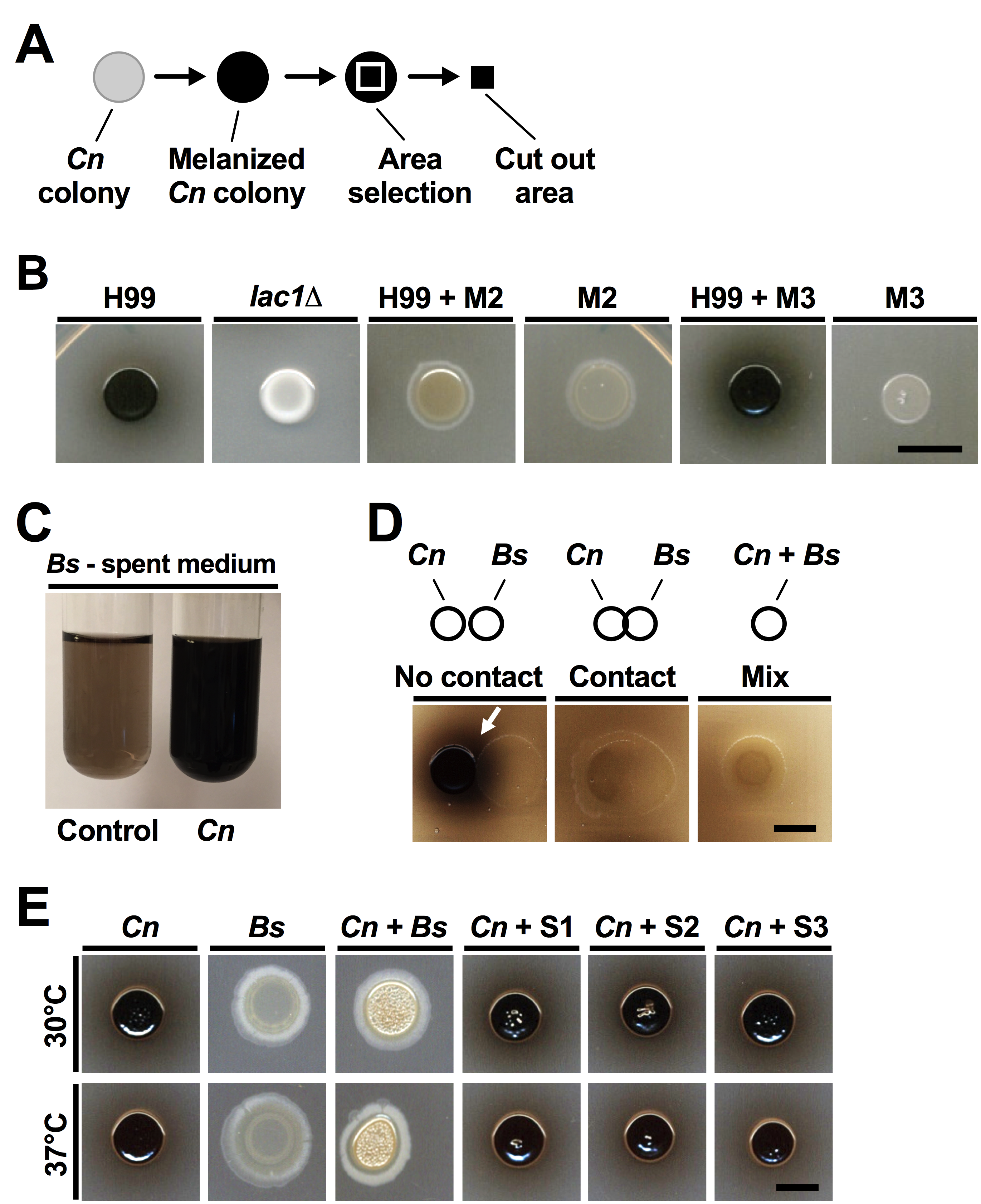

Supplement: FIG S2 [file mbo005173512sf2.tif]

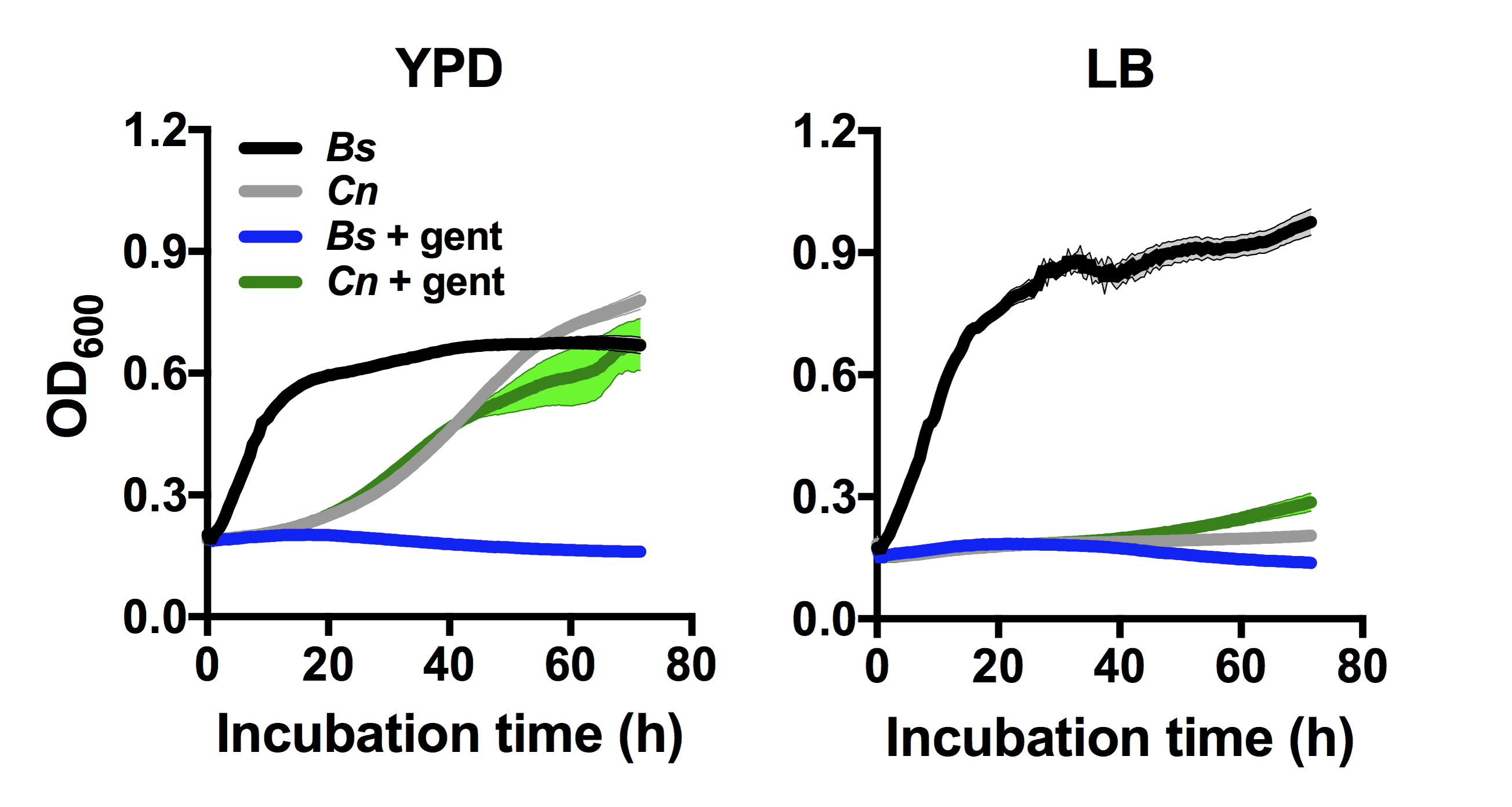

Supplement: FIG S3 [file mbo005173512sf3.tif]

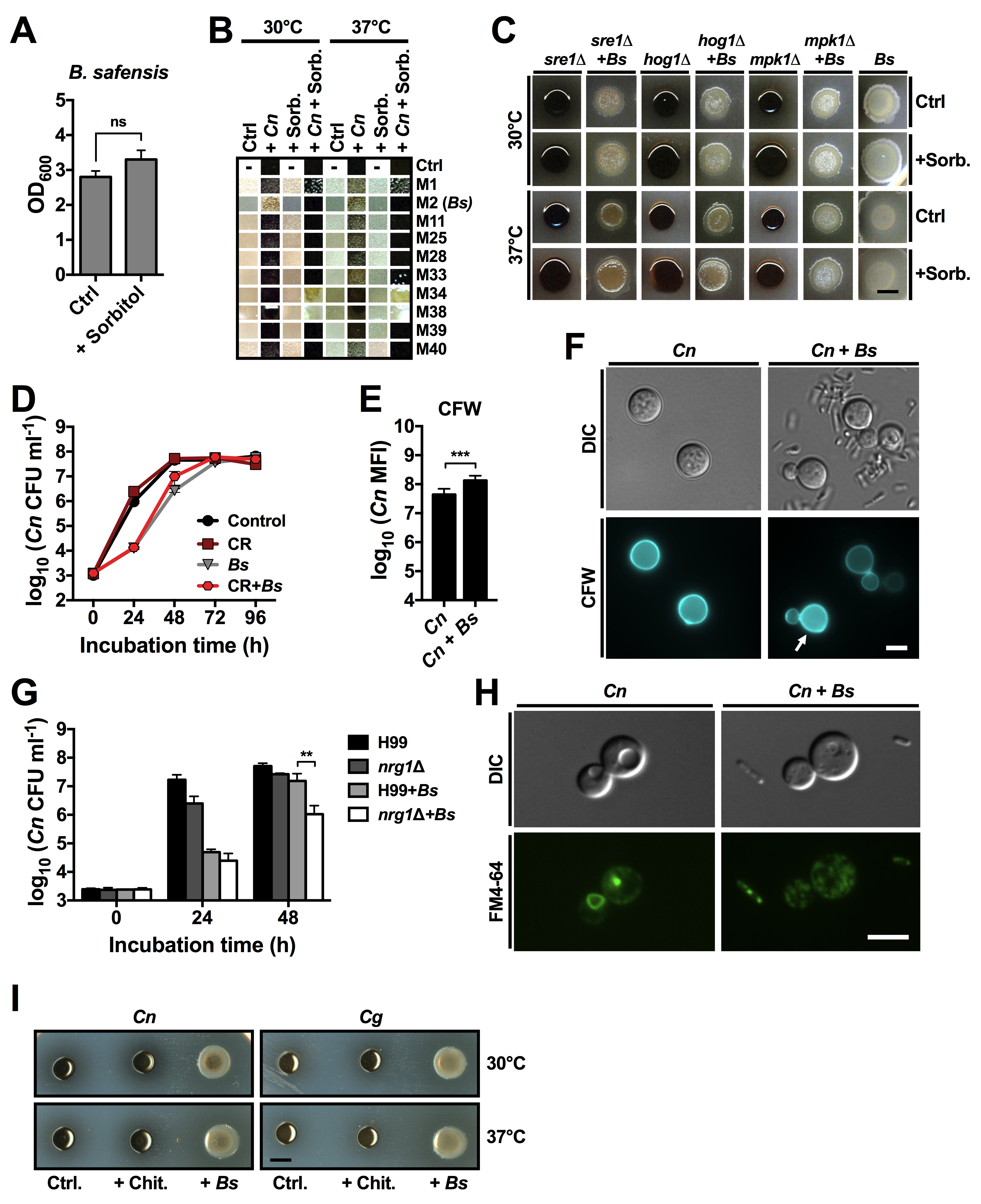

Supplement: FIG S4 [file mbo005173512sf4.tif]

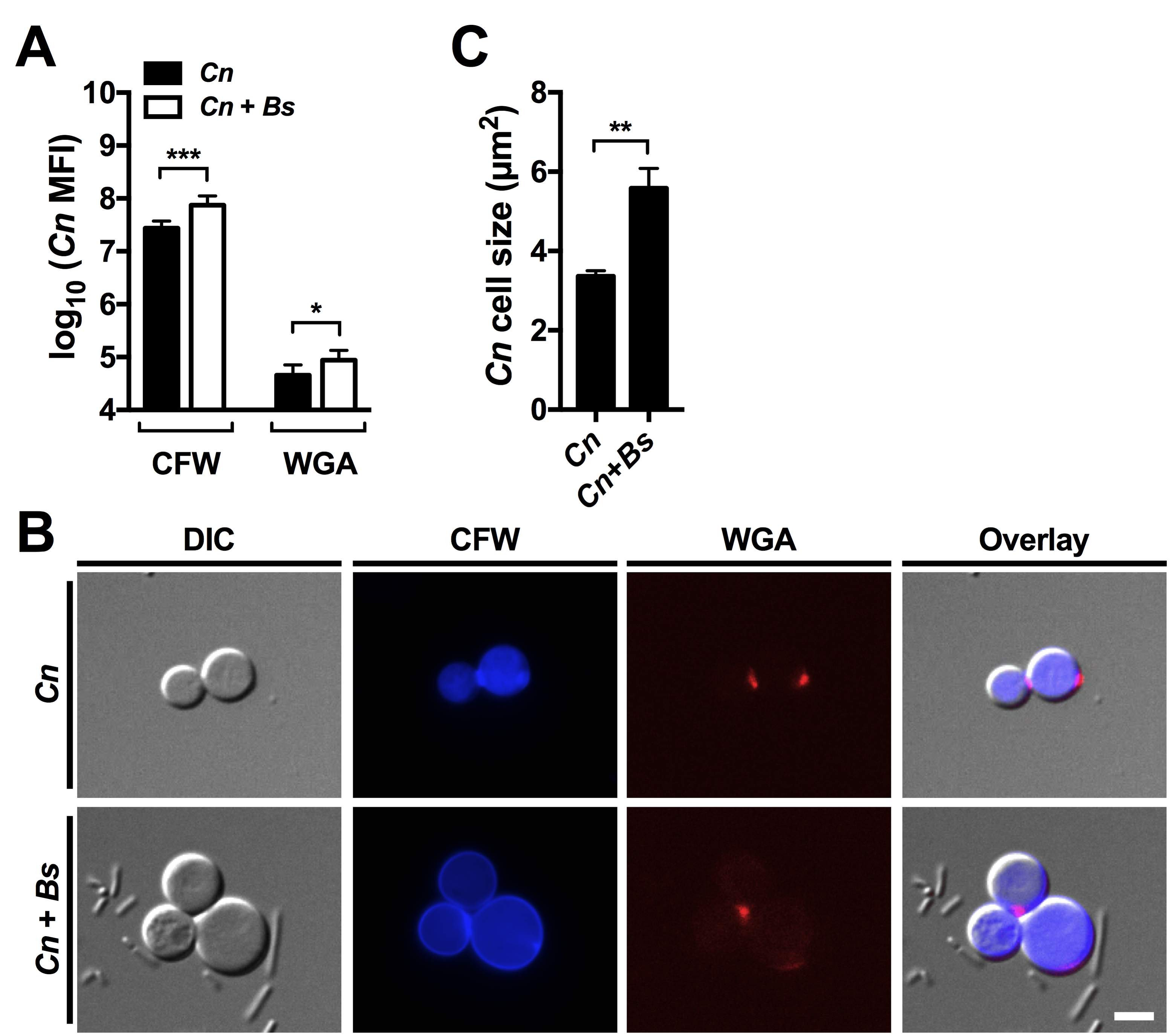

Supplement: FIG S5 [file mbo005173512sf5.tif]

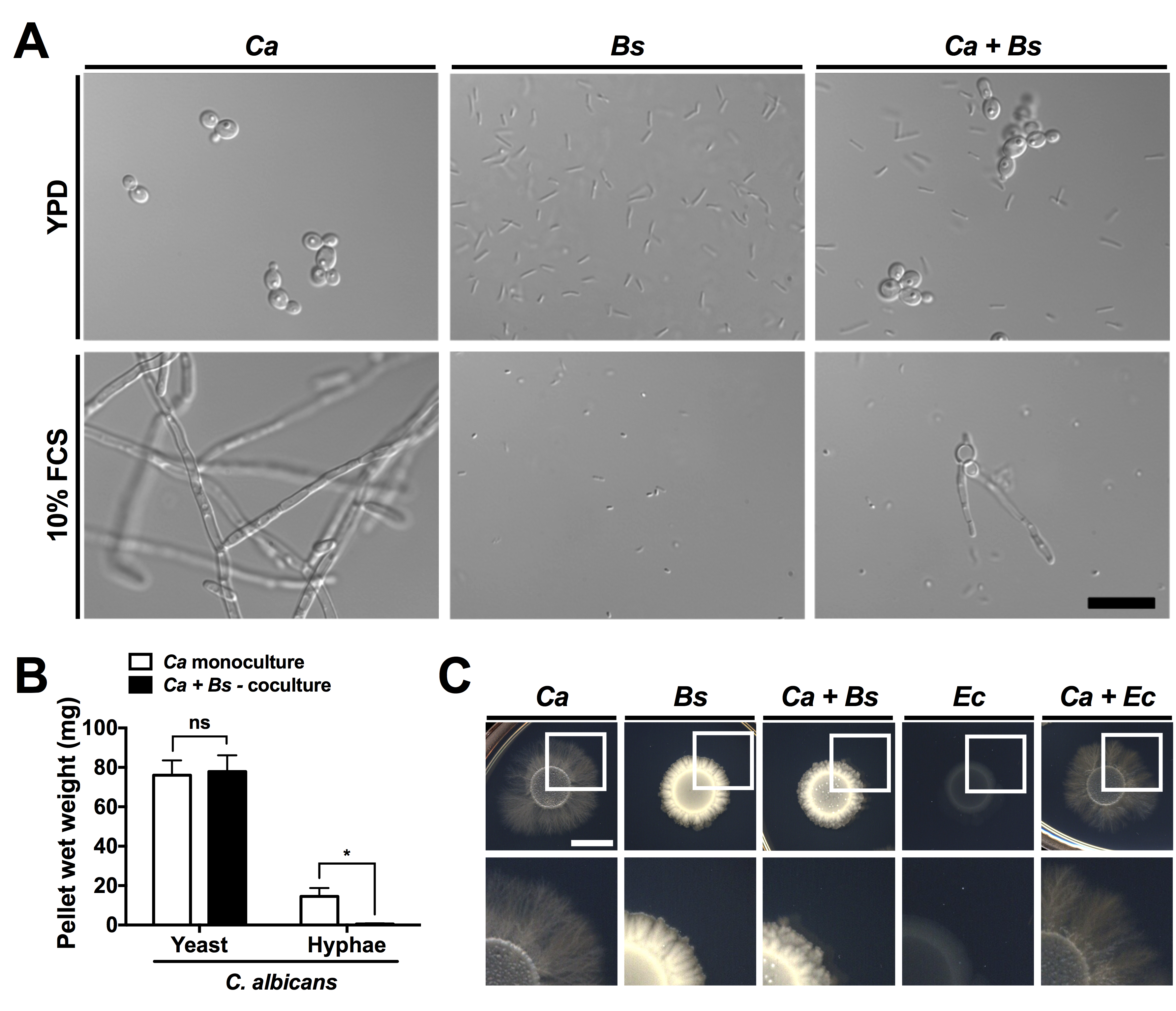

Supplement: FIG S6 [file mbo005173512sf6.tif]

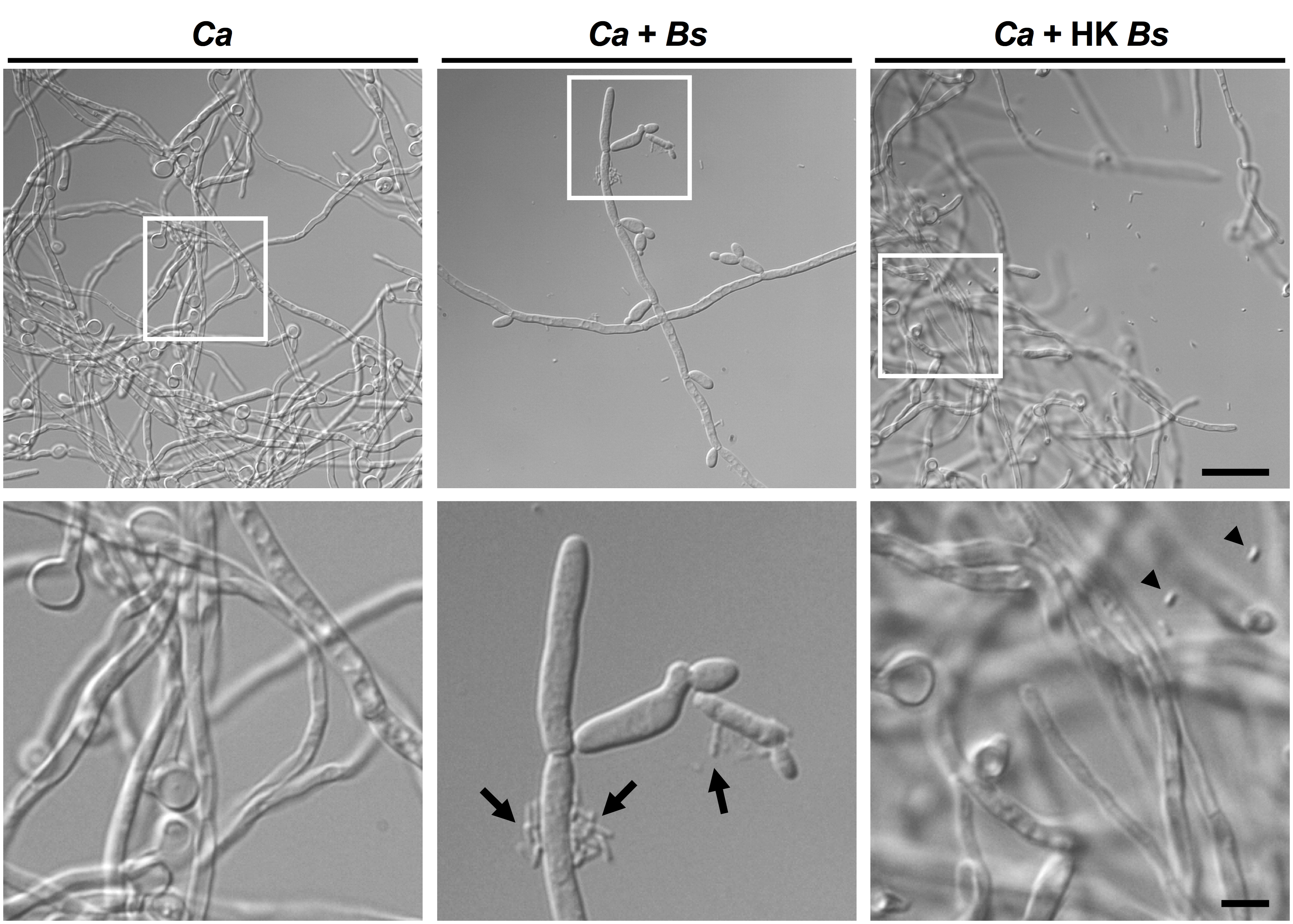

Supplement: FIG S7 [file mbo005173512sf7.tif]

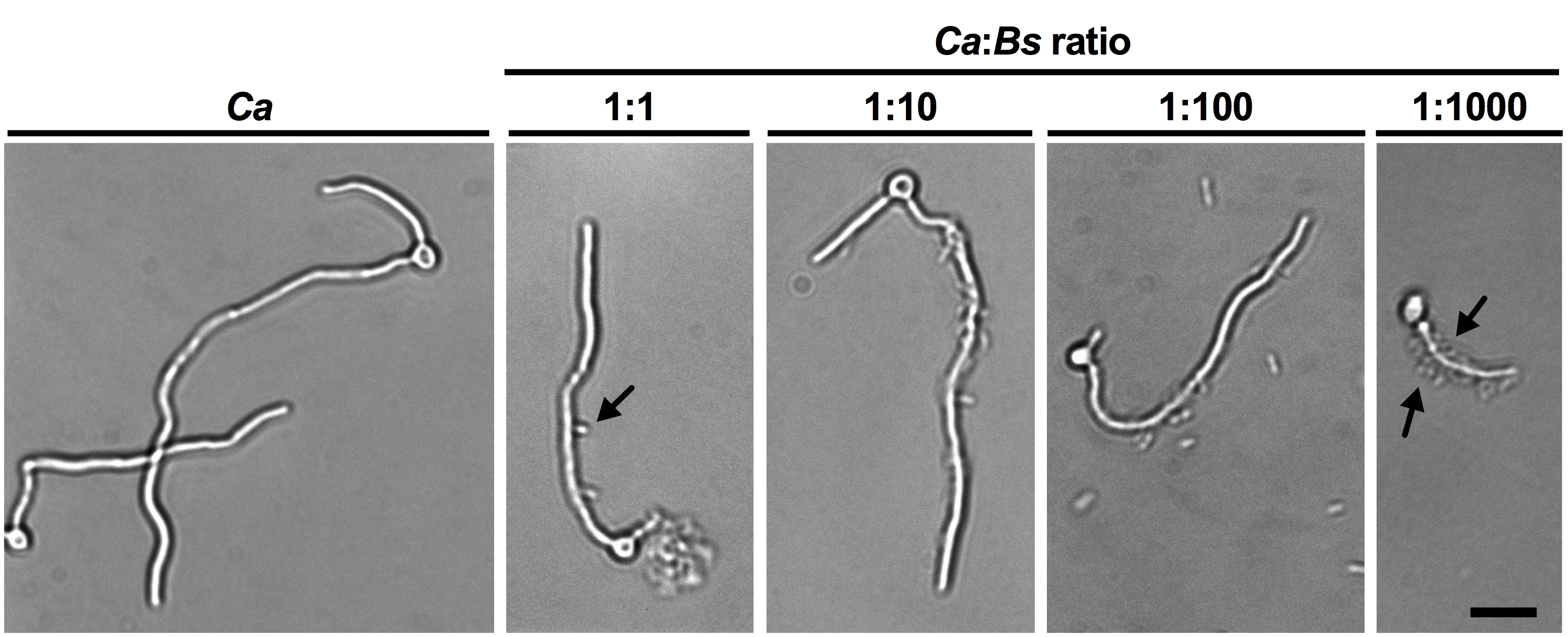

Supplement: FIG S8 [file mbo005173512sf8.tif]
