# Supplementary material for: Disarming Fungal Pathogens: Bacillus safensis Inhibits Virulence Factor Production and Biofilm Formation by Cryptococcus neoformans and Candida albicans
Source: mBio. 2017 Oct 3;8(5):e01537-17. doi: 10.1128/mBio.01537-17 (PMC5626971; doi:10.1128/mBio.01537-17)
Supplement: TABLE S1 [file mbo005173512st1.docx]

**Table S1:** Environmental microbes isolated in this study.

| **Strain**  **ID** | **Microbial species / closest match^a^** | **Max. identity, primer**  **8F** | **Max. identity, primer 1492R** | **Origin** | **Sampling location** |
| --- | --- | --- | --- | --- | --- |
| M1* | *Bacillus megaterium* strain ATCC 14581 | 99% | - | Plant leaf  (*A. japonica*) | UBC campus, Vancouver |
| M2* | *Bacillus safensis*  strain NBRC 100820 | - | 100% | Plant leaf  (*A. japonica*) | UBC campus, Vancouver |
| M3 | n.d. | - | - | Plant leaf  (*U. dioica*) | UBC campus, Vancouver |
| M4 | n.d. | - | - | Soil | Campbell River, Vancouver Island |
| M5 | n.d. | - | - | Soil | Campbell River, Vancouver Island |
| M6 | n.d. | - | - | Soil | Englishman River, Vancouver Island |
| M7 | n.d. | - | - | Soil | Englishman River, Vancouver Island |
| M8 | n.d. | - | - | Soil | Englishman River, Vancouver Island |
| M9 | n.d. | - | - | Soil | Englishman River, Vancouver Island |
| M10 | n.d. | - | - | Soil | Galiano Island, Vancouver Island |
| M11* | *Bacillus aryabhattai*  strain B8W22 | 100% | - | Soil | Hornby Island, Vancouver Island |
| M12 | n.d. | - | - | Soil | Hornby Island, Vancouver Island |
| M13 | n.d. | - | - | Soil | Hornby Island, Vancouver Island |
| M14 | n.d. | - | - | Soil | Qualicum, Vancouver Island |
| M15 | n.d. | - | - | Soil | Qualicum, Vancouver Island |
| M16 | n.d. | - | - | Soil | Qualicum, Vancouver Island |
| M17 | n.d. | - | - | Soil | Qualicum, Vancouver Island |
| M18 | n.d. | - | - | Soil | Qualicum, Vancouver Island |
| M19 | n.d. | - | - | Soil | Qualicum, Vancouver Island |
| M20 | n.d. | - | - | Soil | Qualicum, Vancouver Island |
| M21 | n.d. | - | - | Soil | Qualicum, Vancouver Island |
| M22 | n.d. | - | - | Soil | Qualicum, Vancouver Island |
| M23^#^ | *Paenibacillus xylanexedens* strain B22a | 100% | - | Soil | Qualicum, Vancouver Island |
| M24 | n.d. | - | - | Soil | Qualicum, Vancouver Island |
| M25* | *Bacillus aryabhattai*  strain B8W22 | 99% | - | Soil | Rathtrevor, Vancouver Island |
| M26^#^ | *Bacillus thuringiensis* strain NBRC 101235 | 99% | - | Soil | Rathtrevor, Vancouver Island |
| M27 | n.d. | - | - | Soil | Rathtrevor, Vancouver Island |
| M28* | *Bacillus aryabhattai*  strain B8W22 | 100% | - | Soil | Sproat Lake, Vancouver Island |
| M29 | n.d. | - | - | Soil | Sproat Lake, Vancouver Island |
| M30 | n.d. | - | - | Soil | Sproat Lake, Vancouver Island |
| M31 | n.d. | - | - | Soil | Duncan, Vancouver Island |
| M32^#^ | *Bacillus mycoides*  strain NBRC 101228 | 100% | - | Soil | Duncan, Vancouver Island |
| M33* | *Bacillus aryabhattai*  strain B8W22 | 99% | - | Soil | Duncan, Vancouver Island |
| M34* | *Bacillus subtilis* | 99% | - | Soil | Duncan, Vancouver Island |
| M35^#^ | *Pantoea theicola*  strain QC88-366 | 98% | - | Soil | Victoria, Vancouver Island |
| M36^#^ | *Pantoea theicola*  strain QC88-366 | 98% | - | Soil | Victoria, Vancouver Island |
| M37 | n.d. | - | - | Soil | Victoria, Vancouver Island |
| M38* | *Bacillus subtilis* | 99% | - | Soil | Victoria, Vancouver Island |
| M39* | *Bacillus aryabhattai*  strain B8W22 | 100% | - | Soil | Victoria, Vancouver Island |
| M40* | *Bacillus aryabhattai*  strain B8W22 | 99% | - | Soil | Victoria, Vancouver Island |

^a^, all 10 microbes with (*), and five randomly selected microbes without (#) anti-melanization activity were identified by 16S rRNA sequencing using universal primers 8F and/or 1492R. ~700 bp gene sequence was compared with reference 16S rRNA gene sequences by BLAST-analysis at the National Center for Biotechnology Information (NCBI) website. n.d., not determined; *sp*., species.
