# Supplementary material for: Disarming Fungal Pathogens: Bacillus safensis Inhibits Virulence Factor Production and Biofilm Formation by Cryptococcus neoformans and Candida albicans
Source: mBio. 2017 Oct 3;8(5):e01537-17. doi: 10.1128/mBio.01537-17 (PMC5626971; doi:10.1128/mBio.01537-17)
Supplement: TABLE S2 [file mbo005173512st2.docx]

**Table S2:** Fungal and bacterial strains used in this study.

| **Organism** | **Strain** | **Genotype** | **Source, Reference(s)** |
| --- | --- | --- | --- |
| *C. neoformans* | H99 | Serotype A, *MATα* | Joseph Heitman, USA ([1](#_ENREF_1)) |
|  | *hog1*∆ | *hog1*::*nat* | Hiten Madhani, USA |
|  | *mpk1*∆ | *mpk1*::*nat* | Hiten Madhani, USA |
|  | *sre1*∆ | *sre1*::*nat* | Yong-Sun Bahn, Korea ([2](#_ENREF_2)) |
|  | *nrg1*∆ | *nrg1*::*neo* | Andrew Alspaugh, USA ([3](#_ENREF_3)) |
|  | *lac1*∆ | *lac1*::*hyg* | Jennifer Lodge, USA ([4](#_ENREF_4)) |
|  |  |  |  |
| *C. gattii* | R265 | VGIIa | Karen Bartlett, Canada ([5](#_ENREF_5), [6](#_ENREF_6)) |
|  |  |  |  |
| *C. albicans* | SC5314 | wild-type | Malcolm Whiteway, Canada ([7](#_ENREF_7)) |
|  |  |  |  |
| *E. coli* | DH5α | - | Lab stock |
|  |  |  |  |
| *B. safensis* | M2 | - | This work |

**References**

1. Janbon G, Ormerod KL, Paulet D, Byrnes EJ, 3rd, Yadav V, Chatterjee G, Mullapudi N, Hon CC, Billmyre RB, Brunel F, Bahn YS, Chen W, Chen Y, Chow EW, Coppee JY, Floyd-Averette A, Gaillardin C, Gerik KJ, Goldberg J, Gonzalez-Hilarion S, Gujja S, Hamlin JL, Hsueh YP, Ianiri G, Jones S, Kodira CD, Kozubowski L, Lam W, Marra M, Mesner LD, Mieczkowski PA, Moyrand F, Nielsen K, Proux C, Rossignol T, Schein JE, Sun S, Wollschlaeger C, Wood IA, Zeng Q, Neuveglise C, Newlon CS, Perfect JR, Lodge JK, Idnurm A, Stajich JE, Kronstad JW, Sanyal K, Heitman J, Fraser JA, et al. 2014. Analysis of the genome and transcriptome of *Cryptococcus neoformans* var. *grubii* reveals complex RNA expression and microevolution leading to virulence attenuation. PLoS Genet 10:e1004261.

2. Jung KW, Yang DH, Maeng S, Lee KT, So YS, Hong J, Choi J, Byun HJ, Kim H, Bang S, Song MH, Lee JW, Kim MS, Kim SY, Ji JH, Park G, Kwon H, Cha S, Meyers GL, Wang LL, Jang J, Janbon G, Adedoyin G, Kim T, Averette AK, Heitman J, Cheong E, Lee YH, Lee YW, Bahn YS. 2015. Systematic functional profiling of transcription factor networks in *Cryptococcus neoformans*. Nat Commun 6:6757.

3. Cramer KL, Gerrald QD, Nichols CB, Price MS, Alspaugh JA. 2006. Transcription factor Nrg1 mediates capsule formation, stress response, and pathogenesis in *Cryptococcus neoformans*. Eukaryot Cell 5:1147-56.

4. Missall TA, Moran JM, Corbett JA, Lodge JK. 2005. Distinct stress responses of two functional laccases in *Cryptococcus neoformans* are revealed in the absence of the thiol-specific antioxidant Tsa1. Eukaryot Cell 4:202-8.

5. Kidd SE, Guo H, Bartlett KH, Xu J, Kronstad JW. 2005. Comparative gene genealogies indicate that two clonal lineages of *Cryptococcus gattii* in British Columbia resemble strains from other geographical areas. Eukaryot Cell 4:1629-38.

6. Fraser JA, Giles SS, Wenink EC, Geunes-Boyer SG, Wright JR, Diezmann S, Allen A, Stajich JE, Dietrich FS, Perfect JR, Heitman J. 2005. Same-sex mating and the origin of the Vancouver Island *Cryptococcus gattii* outbreak. Nature 437:1360-4.

7. Gillum AM, Tsay EY, Kirsch DR. 1984. Isolation of the *Candida albicans* gene for orotidine-5'-phosphate decarboxylase by complementation of *S. cerevisiae* *ura3* and *E. coli pyrF* mutations. Mol Gen Genet 198:179-82.
